# Dream content influences daily spirituality

**DOI:** 10.3389/fpsyg.2025.1575174

**Published:** 2025-07-28

**Authors:** John Balch, George Hodulik, Rachel Raider, Aidan David, Chanel Reed, Wesley J. Wildman, David Rohr, Patrick McNamara

**Affiliations:** ^1^Department of Psychology, National University, San Diego, CA, United States; ^2^Center for Mind and Culture, Boston, MA, United States; ^3^Boston University Faculty of Computing and Data Sciences, Boston, MA, United States; ^4^Boston University School of Theology, Boston, MA, United States; ^5^Boston University School of Medicine, Boston, MA, United States

**Keywords:** supernatural agents, dreams, REM sleep, portable EEG devices, religious cognition

## Abstract

**Introduction:**

The daily effects of supernatural-agent (SA) concepts on spirituality remain poorly understood. In this study, we focused on dreaming as an avenue to study the effect of SA concepts on spirituality. This work contributes to a long history of research linking together dreaming and spirituality by utilizing quantitative and longitudinal methods.

**Methods:**

We conducted an intensive longitudinal study of sleep and dreaming among *N* = 124 healthy adults over 2 weeks, with *N* = 61 wearing the Dreem 3 EEG headband to measure sleep architecture We collected dream reports and assessed supernatural content, dream affect, and dreamer agency. Linear mixed effects modeling examined relationships between dream variables and daily spirituality measures To evaluate our time-series data, we constructed a temporal neural network (TSANN) to test causal lagged relationships between our dream predictors and daily spirituality measures.

**Results:**

Dreams containing supernatural content were associated with reduced dreamer agency and more negative affect, and were rated as more bizarre, strange, and scary. Mixed effects models demonstrated that dream affect and REM sleep percentage significantly predicted next-day closeness-to-God ratings and authoritarian God concepts when controlled for participant variance in trait spirituality, as well as effects at a 4-day lag for dream agency and dream affect. The neural network analysis established causal support for the lagged closeness-to-God mixed effects models, with saliency maps showing that 3-4 day lagged predictors influenced model outputs more than 1–2 day lags, demonstrating the importance of multi-day effects in measuring the impact of dream variables on daily closeness-to-God ratings.

**Discussion:**

These findings indicate that SA concepts in dreams contribute to daily levels of spirituality both the following day and with a multi-day lag. We conclude that dreams thus represent a key pathway for the influence of SA concepts on spirituality, and provide a valuable area of study for future research in the psychology of religion.

## 1 Introduction

Billions of people worldwide identify themselves as religious. Religiosity varies significantly across individuals and is strongly related to a range of mental and physical health outcomes in these individuals (Davis et al., 2021; VanderWeele, 2017). Lynn and Schell note that religion and spirituality (R/S) constitute a major influence on human biological variation as well as individual reproductive success (Lynn and Schell, 2024). Despite both the ubiquity of religion in human cultures around the world, as well as a steadily growing literature on the scientific study of religious cognition (see reviews in McNamara, 2022; Van Elk and Aleman, 2017), many aspects of religious belief and behavior remain poorly understood, particularly the cognitive mechanisms that facilitate perception of supernatural agents (SAs) or development and maintenance of a basic God image (Sosis et al., 2017).

God images/SA concepts often operate as a central organizing feature of an individual's R/S experiences and fundamental life narratives (Davis et al., 2013). If the image of God is of a loving, kind, and compassionate figure, then R/S experiences and narratives will on average be positive. Conversely, a harsh, judgmental, and authoritarian image of God will tend to generate greater spiritual struggles, guilt, and anxiety. There is now solid evidence that people's conception of God dramatically influences mental and physical health as well as a variety of wellbeing outcomes (Dezutter et al., 2010). Mencken et al. (2009) present data that suggests that belief in a loving, forgiving God can build bonds of social trust, while belief in a judgmental, authoritarian God tends to make believers more wary of others. Froese and Bader (2008) show that a harsh, judgmental, authoritarian image of God is negatively related to political tolerance and positively related to support for gun ownership and defense spending. However, belief in a morally-interested deity, even a deity who punishes bad behavior, can catalyze social cooperation and basic trust (the so-called “Santa Clause Effect”), presumably because of the possibility of divine surveillance and punishment (Johnson, 2016). Cheating in a social cooperation context becomes more difficult if you are being watched for instances of cheating. In addition to the stable concepts of a personal God image, Sharp and Johnson (2020) and Wilt and Exline (2022) found that the daily felt sense of closeness to God, whether God was morally concerned or not, was an important predictor of theological orientation and the sense of God's contribution to daily social events.

Overall, this body of research has demonstrated that images of God are multidimensional, relational, dynamic, and richly connected to self-perception and daily social outcomes. Given these well-established associations between God image, wellbeing, and social behaviors, it is important to study cognitive mechanisms associated with the generation, maintenance, updating and causal effects of these basic God images. In this paper we provide the first controlled test of one of the oldest existing hypotheses on the origins of God images or SA ideas—namely that they originate in dreams (see Mageo and Sheriff, 2021; McNamara and Bulkeley, 2015). Dreams are a reasonable place to look for some of the physiological and cognitive mechanisms that facilitate SA cognitions. By “originating in dreams” we are not hypothesizing that dreams are the evolutionary source of god concepts or SAs. We see that hypothesis as reasonable but first we sought to establish in this work whether we can observe and measure statistically reliable associations between dream images/content and measures of spiritual and religious experience/cognition. To our knowledge there has yet been a controlled study adequately testing the hypothesis that reliable associations exist between selected measures of dream content and objective daytime measures of spirituality and religious experience (although see Schredl and Mönch, 2023 for a recent interesting cross-sectional study).

Dreams are a reasonable place to look for some of the physiological and cognitive mechanisms that facilitate SA cognitions. Most recalled dreams likely come from “rapid eye movement” or REM sleep. Brain mechanisms associated with REM dreaming are consistent with production of mental simulations of counterfactual, bizarre, and extraordinary images and ideas. During REM dreaming, external sensory input is reduced and the visual centers are activated. Cholinergic and dopaminergic activity levels are high, while serotonergic and noradrenergic levels are low or absent, thus enhancing brain plasticity and pushing the brain-mind into a visually and emotionally driven hyper-associative state (Llewellyn, 2013). In addition, brain regions are dynamically modulated during REM sleep, such as the default mode network (DMN; Fox et al., 2005), the hippocampal–amygdala complex (Maquet et al., 1996), and the ventromedial-prefrontal cortex, while the dorsolateral prefrontal cortex is downregulated (Hobson et al., 2000), a network activation pattern which promotes intense socio-emotional associative processing largely in the absence of reflective thought. Thus, encounters with extraordinary beings in extraordinary realms are likely promoted and accepted uncritically in dreams.

In addition, there is accumulating evidence that REM sleep facilitates consolidation of emotionally significant social events/memories (Stickgold and Walker, 2007). Indeed, most REM dreams depict or simulate social interactions of various kinds (McNamara et al., 2010). Dreams are typically “about” social interactions that involve the dreamer interacting with two to four other characters, about half of whom can be recognized as familiar characters in the dreamer's immediate social network (Domhoff and Schneider, 2018; Tuominen et al., 2022). The other 50% of characters in dreams are unfamiliar to the dreamer and can be overt SAs or vaguely conceived threatening strangers. These kinds of data have prompted investigators to advance the so-called social simulation hypothesis of dreaming (Tuominen et al., 2019), which asserts that most dreams are, among other things, about processing information around important social interactions—including, perhaps, interactions with SAs (Paquette, 2018).

We define SAs as minded agents who exhibit powers beyond those of an ordinary human agent and are considered gods, deities, angels, demons, faeries, ghosts, elves, etc. by the individual experiencing them or by the surrounding culture of the individual. Previous non-experimental descriptive investigations (McNamara, 2016; McNamara et al., 2018; Nordin, 2024a,b) suggest that SAs appear in dreams and interact with the dreamer when there is an associated reduction in the sense of cognitive agency or capacity/power of the dreamer to act effectively in the dream. Once this reduction in dreamer agency occurs there then follows a transfer of cognitive agency (called the “self model” in Nordin's studies) from the dreamer to a different but unfamiliar and salient character who then (frequently, though not invariably) assumes supernatural forms of agency and power in the dream. Because REM sleep is associated with both periodic bodily movements and fluctuating activation levels in midline and ventromedial and supplementary motor cortex (sites associated with the sense of agency, Macrae et al., 2004; Northoff and Bermpohl, 2004), the sense of dreamer agency itself naturally fluctuates during REM dreaming. The study by McNamara et al. (2018) found that these dynamics are most clearly seen in the case of bad dreams or nightmares. Diminished agency within the dreamer occurred in over 90% of nightmares and unpleasant dreams *that had overt SAs* and rarely occurred without SAs. In some two-thirds of unpleasant dreams the SA emerged from an unfamiliar human character. These patterns in the ways in which SAs appear and act in dreams is consistent with rules of character transformation in dreams in general (Rittenhouse et al., 1994) and suggests that there may be a principled way to study their cognitive dynamics in dreams.

In this study we attempted to study the cognitive dynamics of God images and supernatural concepts, realms and SAs in dreams. In study 1 we studied the statistical associations between supernatural content in dreaming and God images/spirituality ratings in waking life across a 2-week period, collecting daily/nightly sleep architectural and dream recall events in the home.

Building on previous research on the dynamism of God images over time, we also tested for lagged associations between the dream content variables and changes in waking God images and concepts. It is difficult when dealing with longitudinal data to identify the “optimal” lag that balances conservative resistance to adding excess variables against losing potentially important temporal dynamics. To address this issue we present a novel approach in study 2 using neural network computational modeling to test the temporal associations we found in study 1.

Our key hypotheses were that 1) supernatural content (defined under methods) in dreams is reliably associated with spirituality ratings and god images in waking life and that 2) selected dream content features (e.g., dreamer agency) are significantly associated with wakingdaily religious or spiritual experiences.

## 2 Study 1

### 2.1 Study 1 methods

We recruited volunteers who answered online adverts for a study on sleep, dreams, nightmares, and religion/spirituality. To be eligible, participants were required to: be at least 18 years old, reside in the US, speak and read English, have reliable Wi-Fi, not have a current psychiatric or neurological diagnosis, and not have a current diagnosis of a sensitive skin condition (to avoid possible skin reactions with the portable EEG device). Volunteers (*N* = 124) were invited to participate in a 2-week study in the home, contributing a maximum of 14 days and nights of observations of daily stress, mood, social activities, sleep and dream measures described below. Since this was a remote study, participants were not based in a single location but widely distributed across the United States. Recruitment was done using the study website as well as social media sites like Facebook. Surveys were distributed online using Qualtrics, with unique identifying codes assigned upon completion of the baseline, enabling researchers to track participants confidentially during the longitudinal portion of the study. Participants were instructed to complete the night surveys right before going to bed and the morning surveys right when they woke up and were permitted to complete the surveys on either their phone or computer. Participants were compensated $200 if they completed the full 2 weeks of participation.

Approximately half of these participants (randomly assigned, *N* = 61) wore the Dreem 3 Headband (DH) designed to derive sleep architecture measures for the duration of the study. They were taught how to start the recording when they were ready to start falling asleep and how to stop the recording when they were ready to wake up in the morning as well as the procedure for charging and ensuring the data successfully transfers. After the training they were instructed to wear the DH that night for habituation and to be sure they were wearing it in the best position for a quality recording. If the channel quality was extremely poor on multiple sensors, participants were asked to adjust the fit for another trial night. Once an acceptable recording quality was achieved, participants began the intensive longitudinal portion of the study, wearing the DH for up to 14 consecutive nights. We collected at least 7 nights of high-quality recordings from 61 participants for a total of 707 usable recordings (see the Supplementary materials for full description of removed data).

#### 2.1.1 Measures of god image and closeness-to-god ratings

##### 2.1.1.1 The limitless, authoritarian, mystical, benevolent, and ineffable measure of god representations scales

The Limitless, Authoritarian, Mystical, Benevolent, and Ineffable Measure of God Representations (LAMBI; Johnson et al., 2019) is a 5-factor questionnaire that asks participants to rate how well different adjectives describe how they view a higher power. This measure was designed to deal with diversity in theistic concepts both within and between religious communities. In order to measure shifting conceptions of God on a day-to-day basis, we used an abbreviated version of the LAMBI scale that used a randomized reduced list of adjectives within each domain for participants to fill out each morning.

##### 2.1.1.2 Brief multidimensional measure of religiosity/spirituality

Participants also completed the BMMRS (Fetzer Institute, 1999). The BMMRS was developed in the late 1990s by a panel of experts in the scientific study of religion who were asked by the Fetzer Institute and the National Institute on Aging to develop an assessment tool that would capture the multidimensional nature of the construct of religiosity. Their primary aim was to develop items for assessing health-relevant domains of religiousness and spirituality. The BMMRS captures 12 domains of spirituality/religion: Daily Spiritual Experiences, Meaning, Values/Beliefs, Forgiveness, Private Religious Practices, Religious/Spiritual Coping, Religious Support, Religious/Spiritual History, Commitment, Organizational Religiousness, Religious Preference, and Overall Self-Ranking as a religious/spiritual person.

Johnstone and Yoon (2009) recently examined the factor structure of the BMMRS in a sample of patients with diverse medical conditions and identified six subdomains underlying eight BMMRS scales: Positive Spirituality, Religious Practices, Forgiveness Cultural Practices, Positive Congregational Support, and Negative Congregational Support. We followed Johnstone and Yoon's method in constructing new variables based on these subdomains, which includes reverse-coding and scaling items to get the same scale and directionality. Cronbach's α for these factors are Positive Spirituality = 0.90, Religious Practices = 0.83, Positive Congregational Support = 0.53, Forgiveness = 0.66, Negative Congregational Support = 0.64.

##### 2.1.1.3 Revised paranormal belief scale

Because beliefs in SAs have been associated with willingness to endorse paranormal beliefs we administered the RPBS (Tobacyk, 2000), a 23-item questionnaire that asks about a variety of unusual and paranormal beliefs that may or may not be tied to an individual's participation in a religious tradition, such as beliefs in reincarnation or in superstitions. We used Tobacyk's suggested subscales to calculate means with one alteration. TRB-1 “The soul continues to exist although the body may die” was moved to the Spiritualism scale due to improved alphas and conceptual fit. Cronbach's α for these subscales were Traditional Religious Beliefs = 0.90, PSI = 0.86, Witchcraft = 0.90, Superstition = 0.80, Spiritualism = 0.83, Precognition = 0.85.

##### 2.1.1.4 Closeness-to-god ratings

In order to assess whether supernatural content would significantly influence daytime spirituality we asked participants each morning to complete a series of Likert rating scales (rated from −10 to +10) assessing the extent to which they felt close to God or their conception of the deity/ultimate. These sorts of daily spirituality ratings have been used and validated in other studies and correspond with a variety of other religious and psychological outcomes (see Sharp and Johnson, 2020).

#### 2.1.2 Sleep, dream and daily/nightly measures of mood and social interactions

##### 2.1.2.1 Sleep architecture

To collect sleep data from participants in their homes we used the EEG wearable DH device (Beacon, 2024). The DH has been successfully validated against gold standard polysomnography (PSG) and sleep staging scoring experts utilizing healthy volunteer adults. The DH has consistently demonstrated high-reliability identification and estimates of sleep staging metrics against PSG and AASM Board certified sleep stage scoring experts. It has also been used in well over 30 studies across very diverse populations (see the Supplementary materials).

These capacities make the DH a practical tool for sleep studies in the home. Via the DH we measured several sleep architecture variables. These are computed for each night based on the 30-s resolution data provided by the DH including: Total Sleep Time (TST, min), Sleep Onset Latency (SOL, min), Wake After Sleep Onset (WASO, min), and REM, N3, and N2 Sleep (min and % of sleep). We did not derive any further EEG-based measures, such as spectral analysis, for this study as these outputs from the DH have not been as thoroughly validated and scored as its sleep architecture algorithms.

##### 2.1.2.2 Dream collection

Our dream collection efforts included surveys and free reports of dream recall each morning. Each morning participants were asked in the morning survey to report any dreams and then rate the content of their dreams in terms of mood and general themes (see the Supplementary materials for full questions and dream cleaning/processing).

##### 2.1.2.3 Dreamland questionnaire

The Dreamland Questionnaire (Holzinger et al., 2020) asks participants to rate dream content along a variety of adjectival scales, such as “strange vs. familiar,” ranging from −10 to +10, with scores in the negative indicating the “strange” end of the scale with lower scores being more extreme. Dream affect was constructed by taking the mean of the five adjective pairs (Unpleasant/Pleasant, Serious/Happy, Scary/Relaxing, Aggressive/Gentle, Pessimistic/Optimistic) with a Cronbach's Alpha of 95.3%.

##### 2.1.2.4 Cognitive agency or dreamer agency scoring

We predicted that reduced dreamer agency would predict greater amounts of supernatural content and SAs in dreams. Three trained research assistants scored each dream for dreamer agency following methods in scoring agency in life history interviews using a scale ranging from 0 to 4 and following rules described and validated by McAdams (2001). A score of 0 was assigned for low agency when, for example, the dreamer was trapped and unable to assert any agency within the dream, and a score of 4 was assigned when the dreamer had complete control over the dream. A dream was scored as 2 if there was no discernible agency displayed. Raters coded a selection of dreams individually, then came together to compare and discuss codes and reach consensus before individually coding another set of dreams for comparison. In total, 1,598 dreams were scored, and we achieved a reliability of Cronbach's Alpha 0.802 between our three raters.

#### 2.1.3 Sample characteristics

Our total number of participants in Study 1 was 124. Participant completion rates of daily/nightly assessments was 97.5% overall (98% morning surveys completed, 97% night surveys completed). Participants were on average 45.71 years old (SD = 14.93), predominantly female (69.4%) and White (66.9%). Over half of the participants had completed a Bachelor's degree or higher (67.7%), and over half had a household income of over $50,000 a year (65.3%). For the longitudinal sample, the average self-rating for religiosity on a scale of 1 to 4 (1 = Not religious at all, 4 = Very religious) was 2.29 (SD = 1.07). The average self-rating for spirituality, also on a scale ranging from 1 to 4 (1 = Not spiritual at all, 4 = Very spiritual), was 3.09 (SD = 0.85). Both scores came from single items on the BMMRS. Participants came from a variety of religious backgrounds and affiliations, with the three largest categories being “Protestant” (23%), Catholic (22.7%), and “Nothing in Particular (10.4%).

#### 2.1.4 Statistical analyses

The intensive longitudinal design of the study allowed for use of multilevel regression analyses (i.e., mixed-effects regression, random-coefficients modeling, hierarchical linear modeling) to test the primary guiding hypothesis that dream content would influence daily God-images in both relational and conceptual dimensions. Multilevel regression techniques were developed to analyze nested, or hierarchical, data structures. The daily diary data, dream content, and sleep architecture assessments served as repeated measures that are nested within individuals. All longitudinal predictor variables were centered around the participant mean to control for between-subjects differences. To analyze the temporal dynamics of dream effects on updating and influencing relational and conceptual views of God, we also constructed lagged variables. Lags are important for assessing the possibility of delayed effects of variables in a time series as well as for investigating whether concurrent measurements are only the consequences of past effects. We chose a 4 day lag as a median to test for longer range effects of dream experiences while still maintaining a sufficiently high number of observations for statistical testing. The inherent difficulties of choosing an optimal lag, however, and the limitations of adequately assessing the importance of a single or even multi-day lag in multi-level regression led to our adoption of a temporal neural network approach in Study 2. Significance testing was set at p < 0.05 for directional tests.

### 2.2 Study 1 results

#### 2.2.1 Study 1.1 results

In Study 1, an in-the-home longitudinal study, we collected a total of 1,599 dreams from 124 participants in 1,704 surveys (including 574 nights with no dream recall) administered across individual 2-week periods. Participants recalled an average of 0.94 dreams per night (SD = 0.94). Three participants did not recall any dreams. To assess supernatural content in dreams we trained two machine learning classifiers on a large database of dream narratives labeled as supernatural or non-supernatural (Supplementary materials). First, we created a single-label text classification model trained on the dreams with supernatural content in the large dataset. We then trained a separate model utilizing Gradient Boosting Classifier due to its high performance and flexibility with a high variety of predictors and due to finding meaningful linguistic differences between supernatural and non-supernatural narratives. This classifier identified 209 nights (18.48% of nights with dream recall) with features associated with supernatural narratives. We took the union of two classifiers to construct our binary variable for supernatural or supernatural-like occurrences during dream content. Scoring of the predictive model against a hand-coded series of supernatural content in dreams found 78.5% agreement.

We then evaluated the features of supernatural narratives in our longitudinal dataset according to the participant's own ratings of different aspects of dream content. We calculated the difference between the means for this content using univariate mixed linear models while adjusting for participant variability. This enabled us to compare means while factoring in baseline differences between participant ratings. We found significant differences in means across 4 dimensions of dream adjectives, with supernatural dreams appearing more strange, bizarre, scary, and aggressive (Table 1). After adjusting for multiple comparisons using the Benjamini-Hochberg corrections, we confirmed these results for dreams appearing more strange, bizarre, and scary but found that aggression was only marginally significant.

**Table 1:** Summary of univariate mixed effects models predicting differences in dream content ratings.

**Participant rating of dream features with and without supernatural content**				
	**Intercept (no supernatural content present)**	**Estimate (supernatural content present)**	***p***	**adj-p**
Strange/familiar	0.277	−1.996	< 0.001	< 0.001
Bizarre/logical	0.753	−1.896	< 0.001	< 0.001
Unpleasant/pleasant	1.082	−0.160	0.697	0.674
Serious/happy	0.463	−0.580	0.142	0.196
Scary/relaxing	1.127	−0.993	0.014	0.033
Aggressive/gentle	1.544	−0.776	0.034	0.057
Pessimistic/optimistic	1.143	−0.158	0.674	0.678

This table summarizes a series of mixed effects models comparing nights with supernatural dream content (0 = no supernatural content, 1 = with supernatural content) with nights without supernatural dream content. All measures are based on participant ratings utilizing adjective pairs, with negative scores corresponding to the left term and positive scores corresponding to the right term. No recall nights were not included. All models included random intercept terms for participant differences. The estimate (when significant) indicates the average difference from the intercept. Assumptions for each model (including normality and homogeneity of residuals) were assessed via visual inspection of Q-Q plots, histograms, and residual-vs.-fitted plots. Adj-p indicates adjusted p-values following Benjamini-Hochberg Correction.

We then also tested the effect of supernatural content on dreamer agency, based on our previously validated coding of “dreamer agency” occurring during the dream narrative. The mean agency (adjusted for participant variability) for dream narratives was 1.812 (using a the 0–4 scale described above). Using a univariate mixed effects model, we also found that supernatural narratives had a −0.135 significantly lower mean for agency (*p* > 0.0001). This result indicates that low agency is an important dimension in the construction of supernatural narratives even when controlling for individual variability in a dream series.

#### 2.2.2 Study 1.2 results

We next tested the relationship between dream content and God images in morning reports (Tables 2, 3). We performed multilevel regressions and lagged analyses on the longitudinal data using two outcomes that proved to be most reliable and accounted for most of the variance among independent variables—namely daily “closeness-to-God” ratings and daily LAMBI ratings on the authoritarian dimension of the God image inventory. We then tested the effects of the proportion of sleep spent in REM sleep (REM%), measured by the DH, on both of our independent variables. Missing data was handled with listwise deletion per model so the number of participants and total numbers of observations is noted on each model. Model construction was done in a forward-step fashion, beginning with an empty model and adding in variables to test for significance and improvement in the model fit based on our hypotheses [following the suggestions in West et al. (2022)]. For the sake of parsimony, we tested groups of variables (such as the BMMRS subscales) and only retained trait-level variables that were significant at p < 0.05 before adding in longitudinal variables. In the case of closeness-to-God, this meant we retained Spiritualism from the RPBS and the Positive Spirituality factor/subscale from the BMMRS (Supplementary Figure S2), while for the LAMBI-Authoritarian (LAMBI-A) dimensions we only retained Traditional Religious Beliefs from the RPBS (see the Supplementary materials for full model outputs). Diagnostic checks indicated divergence from normality in both the residuals and random effects. Consequently, bootstrapping was employed in all models in study 1.2 to derive more robust confidence intervals for the model parameters.

**Table 2:** Mixed effects linear regression models predicting closeness-to-God.

**Closeness-to-God–morning** ***Model 2.1.1 N** = **1,623 observations/124 participants***							
**Predictor level**		**Estimate**	**2.5_ci**	**97.5_ci**	**SE**	**DF**	**T-stat**
**Level-1**							
	(Intercept)	−6.295	−8.607	−3.982	1.18	109.997	−5.335
**Level-2**							
	Spiritualism	0.577^**^	0.169	0.985	0.208	79.451	2.77
	BMMRS positive spirituality	4.825^***^	3.817	5.833	0.514	92.948	9.383
***Model 2.1.2 N** = **1,066 observations/120 participants***							
		**Estimate**	**2.5_ci**	**97.5_ci**	**SE**	**DF**	**T-stat**
**Level-1**							
	(Intercept)	3.403	2.714	4.092	0.351	93.421	9.682
	Dream affect_w_	0.177^*^	0.033	0.32	0.073	927.424	2.414
	Dream agency_w_	0.029	−0.115	0.172	0.073	927.514	0.39
	Supernatural dream content	−0.066	−0.214	0.082	0.076	949.903	−0.877
	Spiritualism ^*^ dream agency	−0.233^***^	−0.371	−0.096	0.07	927.425	−3.327
	Spiritualism ^*^ dream affect	0.243^***^	0.108	0.378	0.069	927.478	3.537
**Level-2**							
	Spiritualism	0.779	0.164	1.393	0.313	74.896	2.485
	BMMRS positive spirituality	3.484^***^	2.761	4.207	0.369	78.98	9.444
	Dream affect_b_	0.027	−0.627	0.68	0.333	71.038	0.081
	Dream agency_b_	−0.053	−0.732	0.626	0.346	61.118	−0.153
***Model 2.1.3 N** = **756 observations/118 participants***							
		**Estimate**	**2.5_ci**	**97.5_ci**	**SE**	**DF**	**T-stat**
**Level-1**							
	(Intercept)	3.495	2.717	4.212	0.377	87.869	9.281
	Dream affect _w_(T-4)	0.147	−0.001	0.295	0.075	645.63	1.953
	Dream agency_w_ (T-4)	−0.182^*^	−0.332	−0.039	0.075	640.359	−2.442
	Supernatural dream content (T-4)	−0.157^*^	−0.308	−0.003	0.076	651.006	−2.06
**Level-2**							
	Spiritualism	0.789^*^	0.138	1.422	0.328	71.738	2.408
	BMMRS positive spirituality	3.548^***^	2.77	4.385	0.39	75.973	9.092
	Dream affect_b_	−0.169	−0.934	0.583	0.377	74.213	−0.447
	Dream agency_b_	0.195	−0.553	0.897	0.374	59.199	0.522
***Model 2.1.4 N** = **666 observations/61 participants***							
		**Estimate**	**2.5_ci**	**97.5_ci**	**SE**	**DF**	**T-stat**
**Level-1**							
	(Intercept)	3.164	2.128	4.195	0.56	43.315	5.654
	REM%_w_	0.208^**^	0.051	0.368	0.078	604.335	2.674
**Level-2**							
	BMMRS positive spirituality	3.769^***^	2.526	4.984	0.635	40.605	5.933
	Spiritualism	0.959^*^	−0.068	1.9	0.461	29.255	2.079
	REM%_b_	0.026	−0.662	0.759	0.37	18.643	0.072

BMMRS, Brief Multidimensional Measure of Religiosity/Spirituality; REM%, percentage of sleep in REM sleep, as scored by the DH. _w_ subscript indicates a within-subjects term, _b_ subscript indicates a within-subjects term. All continuous predictors were standardized (z-score scaled) prior to analysis. Level-1 indicates a longitudinal-level variable while level-2 indicates a trait-level variable. ^*^p < 0.05, ^**^p < 0.01, ^***^p < 0.001.

**Table 3:** Mixed effects linear regression models predicting authoritarian god concepts.

**LAMBI-authoritarian** ***Model 2.2.1*** **N** = **1,673 observations/124 participants**							
		**Estimate**	**2.5_ci**	**97.5_ci**	**SE**	**DF**	**T-stat**
**Level-1**							
	(Intercept)	3.123	2.907	3.34	0.11	119.302	28.275
Level-2							
	TRB	0.562^***^	0.345	0.778	0.111	119.068	5.081
***Model 2.2.2 N** = **1,109 observations/120 participants***							
		**Estimate**	**2.5_ci**	**97.5_ci**	**SE**	**DF**	**T-stat**
**Level-1**							
	(Intercept)	3.049	2.817	3.273	0.112	116.682	27.215
	Supernatural dream content	0.009	−0.034	0.054	0.023	985.799	0.388
	Dream affect_w_	−0.073^**^	−0.116	−0.025	0.023	970.006	−3.228
	Dream agency_w_	−0.003	−0.049	0.042	0.022	970.08	−0.148
	TRB ^*^ dream affect_w_	0.058^**^	0.019	0.1	0.021	970.009	2.803
**Level-2**							
	TRB	0.529^***^	0.305	0.749	0.115	116.684	4.618
	Dream affect_b_	−0.035	−0.308	0.258	0.137	119.944	−0.259
	Dream agency_b_	−0.178	−0.465	0.114	0.149	116.919	−1.198
***Model 2.2.3 N** = **696 observations/61 participants***							
		**Estimate**	**2.5_ci**	**97.5_ci**	**SE**	**DF**	**T-stat**
**Level-1**							
	(Intercept)	3.204	2.871	3.539	0.164	56.525	19.551
	REM%_w_	0.054^*^	0.007	0.104	0.025	631.618	2.219
	REM%_w_^*^ TRB	−0.053^*^	−0.099	−0.004	0.024	631.618	−2.189
**Level-2**							
	TRB	0.697^***^	0.382	1.011	0.169	56.283	4.113
	REM%_b_	0.483^**^	0.153	0.799	0.16	57.89	3.02
***Model 2.2.4 N** = **783 observations/120 participants***							
		**Estimate**	**2.5_ci**	**97.5_ci**	**SE**	**DF**	**T-stat**
**Level-1**							
	(Intercept)	3.057	2.853	3.273	0.11	116.589	27.766
	Dream affect_w_ (T-4)	0.085^***^	0.034	0.138	0.026	661.359	3.308
	Dream agency_w_ (T-4)	−0.045	−0.093	0.005	0.025	659.463	−1.759
	Supernatural dream content (T-4)	0	−0.05	0.053	0.026	671.039	0.012
	LAMBI-A_w_ (T-4)	0.011	−0.039	0.054	0.024	661.002	0.447
**Level-2**							
	TRB	0.527^***^	0.314	0.742	0.112	116.831	4.714
	Dream affect_b_	0.096	−0.186	0.382	0.139	119.615	0.688
	Dream agency_b_	−0.279	−0.567	−0.002	0.147	117.586	−1.901

LAMBI-A, Authoritarian subdimension of the LAMBI Concepts of God Scales; TRB, Traditional Religious Beliefs; REM%, percentage of sleep in REM sleep, as scored by the DH. _w_ subscript indicates a within-subjects term, _b_ subscript indicates a within-subjects term. All continuous predictors were standardized (z-score scaled) prior to analysis. Level-1 indicates a longitudinal-level variable while level-2 indicates a trait-level variable. ^*^p < 0.05, ^**^p < 0.01, ^***^p < 0.001.

In all models, we included individual participant variability as a random effect, which adjusts the intercepts based on participant variability. Finally, lagged analyses were conducted iteratively to examine the temporal relationship between dream content indices and appearance of supernatural content in dreaming.

### 2.3 Study 1 summary

We found that dreams classified as having supernatural content were rated as more bizarre, strange, and scary by our participants. We found that participants who rated higher on baseline levels of positive spirituality or spiritualism were more likely to endorse overall stronger feelings of closeness-to-God. Dream affect was positively associated with closeness-to-God, while the interaction between dream affect and spiritualism had a negative association, and the interaction between dream agency and spiritualism had a positive association (Supplementary Figure S6). With regard to the second major outcome variable of morning “authoritarian” conceptions of God, these were positively predicted by traditional religious beliefs at the baseline level, and negatively by within-subjects dream affect at the longitudinal level. The lagged analysis assessed the impact of dream content variables on morning ratings from 4 nights ago. Lagged dreamer agency and supernatural dream content were negatively associated with morning closeness-to-God, while lagged dream affect had a similar effect on authoritarian concepts to God as the concurrent measurement. For the sleep architecture variables, morning closeness-to-God and authoritarian concepts of God were positively related to within-persons levels of nightly REM% even when controlling for baseline beliefs, although the effect on closeness-to-God was much stronger. Between-subjects REM% was also significantly positively related to authoritarian concepts of God, but not to closeness. While the lagged analysis points to carry-over effects over time from dreams to God-images and -concepts, mixed effects regression is limited in its capacity to diagnose the causal impact of lagged variables beyond metrics like significance. To account for these issues in time-series data, we developed Artificial Neural Networks (ANNs) to give a more nuanced understanding of the temporal dynamics and interdependencies of the predictors.

## 3 Study 2

### 3.1 Study 2 methods

In study 1, a Linear Mixed Effects (LME) model was employed to analyze different effects on closeness-to-God and LAMBI-Authoritarian God-image ratings while accounting for random effects per participant. While the simplicity of the LME is a strength, LMEs are limited in the complexity of temporal relationships they can represent. Artificial Neural Networks (ANNs), however, have highly customizable architectures that have been used to represent many different kinds of complex temporal relationships. In study 2.1, we first designed an ANN to mimic the functionality of the LME, demonstrating the similarities between these classes of models. Then in study 2.2, we designed an ANN which encapsulates the time-sequential relationship in 4-day data episodes and compared performance to the LME in participant-stratified 3-Fold cross-validation. We call this ANN “Temporally Sequential Artificial Neural Network,” or TSANN. Because ANNs have a reputation of being opaque black-box models, in study 2.2 we applied a technique called *post-hoc* saliency map analysis on TSANN to gain insight on its inner workings. While most often applied to image classification ANN tasks, saliency maps are a *post-hoc* analysis technique that can be applied to many ANN applications as it utilizes model input sensitivities present in the gradient of an ANN (Simonyan et al., 2013). Finally, in study 2.3, we retrained altered versions of TSANN which used shorter 1–3 day time lags. Performance of these versions of TSANN can be compared to each other to learn which time lags allow the model to account for most variance, offering supporting evidence of the causal-temporal relationships in the data, and offering insight into how many lagged days are needed to capture the full time lag effect between dream characteristics and closeness-to-God and LAMBI-Authoritarian God-image.

#### 3.1.1 LME vs. TSANN

An LME model can estimate the effects of dream characteristics on closeness-to-God and LAMBI-Authoritarian scores, accounting for variations between subjects (random effects) and potential correlations over time (via autoregressive structures, for example). However, LMEs assume linear relationships and limited interactions over time—meaning that they could struggle to account for non-linear and highly interdependent effects between, say, the vividness of dreams on Day 1, emotional intensity on Day 3, and the final score on Day 4. In our LME, we limited our explorations of time lags to only include one set of time-lagged data points (e.g., Day T-4 influencing Day T); while we could have given the LME all intermediate time points (e.g., Day T-4, T-3, T-3, T-2, and T-1), at best an LME could account for simple linear relationships on the temporal correlations in the data. For example, even if an LME with intermediate time points showed significant relationships in the data, those relationships are constrained by what can be represented in a linear regression: each variable or interactions would have a calculated significance and effect size that are linearly combined to estimate the dependent variable.

However, the LME cannot utilize subject matter expertise regarding the cumulative and sequential effect of time in these longitudinal data, nor can it account for non-linear relationships. Consider our general hypothesis that dream imagery, dream affect, and agency has an effect on closeness-to-God ratings. An LME can tell us if an effect is there, but it cannot account for nuance in the temporally sequenced data. For example, it is simple enough to consider how a particularly vivid, positive affect, low agency dream could then increase or decrease closeness-to-God ratings one or more days later. But what if the participant has a particularly affective dream on night T-4 and T-3, then mundane dreams on nights T-2 and T-1? Or any combination of dream intensities over prior nights? Does having a certain kind of dream on one of the intermediate days counteract any effect of a vivid dream? An LME is able to consider many types of relationships between these temporal variations, but because it cannot capture a temporal sequence of events, it has to consider these temporal data points relatively equally; an LME may be able to tell us that night T-2 is significant, but it is not aware that night T-2 has special relationships with nights T-1 and T-3 in that they are temporally in sequence with one another. TSANN's architecture deliberately specifies a sequence of computations, first calculating the effect of the oldest time point, then passing that effect on to the next day and so on.

Say Participant A reports a vivid dream on Day 1 and emotional intensity on Day 2, but their closeness-to-God score remains unchanged on those days. On Day 3, they have a less vivid dream, but their emotional intensity score remains high, and on Day 4, they report a sharp increase in their closeness-to-God score. The LME might struggle to capture the delayed effect of the high vividness from Day 1 combined with emotional intensity from Day 2 influencing the Closeness-to-God score on Day 4 because it treats the temporal effects more simplistically.

TSANN, however, would model this interaction across time by propagating the information through its layers, allowing Day1′s vivid dream to influence how the emotional intensity of Day 2 affects Day 3, then Day 4, in a non-linear and cumulative manner. Thus, TSANN better captures these complex temporal and interactional effects, offering insights into causal relationships that are more nuanced than what an LME could detect.

#### 3.1.2 Study 2.1 methods

In this study we designed a simple ANN with one perceptron unit—the basic unit of any ANN—to mimic the LME from study 1. The basic requirement for this network is that it must linearly combine T-4 lagged data and participant random effects to produce our dependent variable estimate. The perceptron unit already linearly combines model inputs; it is the perceptron's activation function that introduces non-linearity into an ANN. So, we can simply omit the activation function to mimic the LME's linear combination. The perceptron unit also typically learns a bias to act as the intercept in the linear combination. In the LME, however, the regression intercept is substituted with the calculated participant random effect. So, we can also omit this bias in the perceptron unit to mimic this function. The final piece in our mimicry of the LME is the calculation of random effects themselves. The LME constrains the random effects of participants to fit a normal distribution, then optimizes on log loss. We extracted the calculated random effects from the LME fit without random slopes or effects, and passed the random effect values directly into the ANN. Alternatively, we can calculate our own random effects by generating a scalar (one dimensional) embedding of the participant-IDs *in-situ*. However, the *in-situ* scalar embeddings do not constrain the participant embeddings. For study 2.1, we compared the study 1 LME to ANNs which use the study 1 LME's random effects vs. calculated *in-situ* embeddings, using R^2^ and Mean Squared Error (MSE) as comparison metrics.

#### 3.1.3 Study 2.2 methods

##### 3.1.3.1 Temporal ANNs overview

In study 2.1, we compared a simple mixed effects ANN against our LME in study 1, demonstrating how an ANN can use mixed effects in a similar manner using unconstrained participant scalar embeddings, and that doing so does not appear to give the model a unilateral advantage/disadvantage in this regard. In this study, we designed a more complex ANN which accounted for the temporal sequential structure of our data, which we call Temporally Sequential ANN, or TSANN. ANNs such as Recurrent Neural Nets (RNNs), Long-Short-Term Memory (LSTM) models, and Temporal Convolutional Networks (TCNs) have a robust history of exploiting temporal structure in data. In brief, RNNs and LSTM models retain a “memory” of temporal history to inform future estimates. TCNs, on the other hand, utilize causal convolutions, where estimates can only access current and past time steps, to encapsulate temporal structure of the data. Mahmoud and Mohammed (2024) goes into much more detail about RNNs, LSTM models, TCNs, and other temporal neural networks. Our TSANN model pulls primarily from the theoretical bases for TCNs and causal convolutions, however it does not directly apply causal convolutions in a strict sense, so it is not accurate to call TSANN a TCN.

##### 3.1.3.2 TSANN design

**Figure 1:**
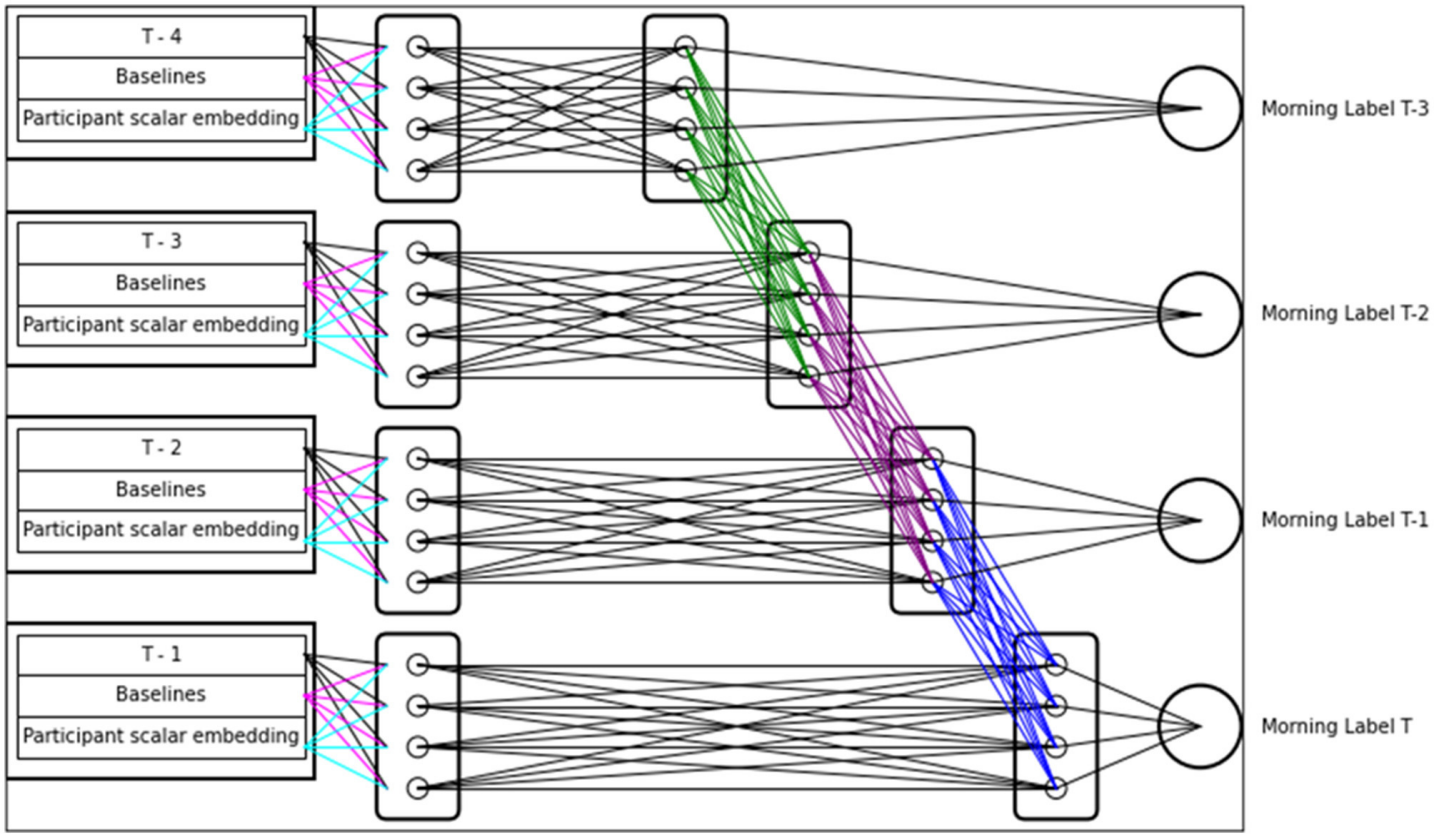
Temporal neural network model. This figure demonstrates the temporal structure of an artificial neural network. Each set of time inputs are passed along to an independent set of nodes, the first hidden layer of TSANN indicated by the four rectangles that the inputs connect to. Outputs from the first hidden layer node sets are passed on to second layer hidden node sets, where the second layer outputs are then both passed to time output perceptrons, as well as the second hidden layer nodes for the subsequent time step. Thus, each output of the model is only fed forward data from past time steps, and the multiple tasks of each time point allow the layers of TSANN to more easily learn generalizable trends in the data. This process is similar to the causal convolutions in Temporal Convolutional Nets (TCNs), which also feed data forward to outputs of future time steps.

Figure 1 diagrams the temporal structure of TSANN. In this section we will walk through the sequence of calculations TSANN performs when generating its estimates. First consider the top row of the diagram, which most closely resembles our ANN from Study 2.1 and our LME. The T-4 data, participant baseline data, and scalar embeddings of this unit are identical in structure to the input for our 3.1 ANN and our LME. To the right of these inputs, four perceptrons are displayed within a larger rectangle. Note that the diagram displays four perceptrons, but the actual number of perceptrons in each rectangle can be controlled by the TSANN hyperparameter N (the number of hidden neurons per node group). The N hyperparameter influences the complexity of the relationships TSANN can represent internally, which means that low N may not fit optimally, and a high N may learn from training data too well and overfit. Each of these perceptrons linearly combines the inputs to the left in a manner identical to the Study 2.1 ANN, with key differences that these perceptrons then pass their outputs through a non-linear activation function known as LeakyReLU, and these perceptrons are also allowed to have their own bias independent of the participant embedding. Since the perceptrons are independent, however, they can each learn their own input weights, each potentially estimating different latent variables. After passing through the activation function, the perceptron outputs pass through another hidden layer of four perceptrons before being processed by the final perceptron labeled “Morning Label T-3,” where a final linear combination and non-linear activation is processed to produce a T-3 prediction. Note that even though we provide all intermediate time points to the model as a whole, the T-3 estimate only “sees” the T-4 data, thus applying a temporal constraint in a very similar manner to a TCN's causal convolution. Each of the next intermediate time data points are similarly passed into a hidden layer of 4 perceptrons, then passed to a second hidden layer. However, as represented by the green, purple, and blue lines, data also flows forward in time. For instance, the T-2 estimate “sees” the T-3 data in the same manner that the T-3 estimate saw T-4 data, but the second hidden layer is also given the outputs of the second hidden layer from the prior time point. Not only does this allow the T-2 estimate to consider the prior days, it also constrains the T-4 second hidden layer to perform calculations that both improve the T-3 estimate, as well as indirectly improving all the future time point estimates. While at face value we may expect such a constraint to hinder model performance, the opposite is often true for ANN Multi-Task Learners (MTLs); this phenomenon is called inductive transfer (Caruana, 1997). When an ANN is learning multiple related tasks at once, the network is often able to share useful information between the tasks and enable the model to learn a shared representation with better generalization. TSANN is inherently an MTL, as it is learning multiple related tasks at the same time.

The last significant element of TSANN is the constraints we placed on weights from baseline data and the participant scalar embedding. In Figure 1, these constraints are represented visually with the magenta and cyan lines. In our LME, baseline inputs and random effects are considered singularly, because the model only has one point of calculation which uses these values. However, TSANN performs a calculation using baselines and participant scalar embeddings once for each perceptron in the first hidden layer. While the baselines and embeddings themselves are constant, each perceptron can learn vastly different weights to apply these inputs. We did not want the model to be able to completely freely assign weights because we make an assumption that each night's data is processed in similar manners before being combined with other night's data. Therefore, we constrain that the weights of baselines and embeddings must be the same for each set of corresponding perceptrons—that is, the top perceptron in the T-4 first hidden layer must treat baselines and embeddings identically to the top perceptron in the T-3, T-2, and T-1 layers. Similarly, the second to top perceptron must have identical weights to all the other second to top perceptrons, and so on. We enforce this constraint by calculating the squared distance between these weights that should be identical (or trivially similar), and add the squared distance to the cost function of the model.

Ultimately, TSANN embeds assumptions about the temporal relationship between data points. The model assumes that each days' data is first independently processed in similar ways (caused by the constraints on baseline and embedding weights), then combined with past data to make a next-day prediction, then is passed on to the next day's data to produce that next-day prediction. This is intended to capture what we assume about dream characteristics' effects on closeness-to-God and LAMBI-Authoritarian ratings: that each night's dream characteristics have an individual effect that is modified by the cumulation of the prior night's dream characteristics. Rather than simultaneously considering all data points at once, as an LME would do, there is a computational structure to TSANN that is much more akin to a causal computational model.

##### 3.1.3.3 Data processing

The LME model focused only on the 4-day lag, using T-4 inputs to predict time T outputs, ignoring data from intermediate times between T-4 and T. The ANN from Study 2.1 mimicked this model design. But TSANN uses T-4, T-3, T-2, and T-1 inputs to predict outputs at time T, T-1, T-2, and T-3. This requires more data points to make up a single training or testing sample, thus more sample removals due to missing data than in the study 1 and study 2.1 cases. To create one sample in our study, a participant must have several consecutive days of complete data for the model input at times T-4 to T-1, and the time T value as a dependent variable. A participant with complete data on the model inputs for the full 14 days of data collection will create 10 entries. But a single missing value can remove up to 5 entries, depending on the time point of the missing data. After creating this data set, we removed participants who had fewer than 3 total entries, as 3 entries or more are required for the participant-stratified 3-fold cross-validation to be valid (see next section). After this reprocessing the morning closeness-to-God condition had 268 entries over 41 participants, and the LAMBI-Authoritarian condition had 311 entries over 47 participants.

##### 3.1.3.4 Analyses

To compare TSANN to the study 1 LME, we used participant-stratified 3-Fold cross-validation. K-fold cross-validation is a statistical technique used to measure a model's ability to respond to novel data. Instead of fitting the model to one set of data and evaluating on the same set of data, K-fold cross-validation splits the data into K random sets called folds. The model is then evaluated K times, each time reserving 1 fold for testing and using the remaining K-1 folds to train the model. An ANN model that performs well on training data but poorly on unseen data is not learning generalizable trends, suggesting that the model is overfit—that is, in thrall to training data and inflexible when encountering unfamiliar data.

Cross-validation gives us a better comparison of models specifically when comparing how much generalizable information the model learns. However, because our models use participant random effects and participant embeddings, we must do participant-stratified cross-validation, meaning that each cross-validation fold must have similar amounts of examples per participant, and at least one example per participant. If a participant were missing from a fold, then the model would not be able to perform when that fold is used as the testing set.

The size of a fold is also the size of a test set during each iteration of the evaluation. In larger data sets, it is more common to use K = 5 or K = 10. However, increasing K also means increasing how many complete entries each participant is required to have to be included in the study. Setting K = 3 balances the need for a reasonable number of entries in the test set and the availability of 5-day data histories.

For study 2.2 analyses, the LME was trained on the same processed data, omitting the intermediate time lagged variables. The training was repeated 30 times, with a different selection of folds each iteration. TSANN was trained on the dataset without omission, and we varied hyperparameter N and training parameter epochs (Supplementary Table 9). Each parameter combination was repeated 30 times, with a different selection of folds each iteration. The best combinations of hyperparameters and training parameters were compared to the LME results using a Welch's *t*-test to account for unequal variances for the LME tests and TSANN tests. Finally, saliency maps on the best variations were performed to measure the relative impacts of different inputs on the different outputs.

#### 3.1.4 Study 2.3 methods

In study 2.2, we assumed a 4-day (T-4) lag was optimal/required to encapsulate the relationship between dream characteristics and closeness-to-God/LAMBI-A. For Study 2.3, we additionally trained modified versions of TSANN for 3-day (T-3), 2-day (T-2), and 1-day (T-1) time lags. Study 2.1 had experiments using a T-4 time lag without the intermediate one- to 3-day time lagged values (Supplementary Figures S3–S5). Study 2.2 used a four-day time lag with intermediate values. Each different time lag is a slight modification to the Study 2.2 model, with time sections of the network clearly removed for lower time lags.

Using the same processed data from Study 2.2, each network in Study 2.3 was cross-validated 200 times for combinations of N and epochs of N: [2, 3, 4, 5, 6] and epochs: [250, 500, 750, 1,000, 1,250, 1,500, 1,750, 2,000] and epochs. We denote the time lag variations by *d*: [4, 3, 2, 1], where *d* denotes the number of days prior to time of measurement T. Note that N only denotes the number of perceptrons per node group. Since there are two node groups per number of lag days *d*, as denoted in T-*d*, the total number of perceptrons P_d_ in each model is calculated as 2 ^*^ *d* ^*^ N. So for P_4_ when *N* = 6 then the total number of perceptrons is 48.

The outputs of the decided combinations are analyzed for assumptions of normality and homogeneity of variance for deviance in variances. Based on these analyses, an appropriate test is applied (e.g., one-way ANOVA, Welch's ANOVA, Krusal-Wallis H).

### 3.2 Study 2 results

#### 3.2.1 Study 2.1 results

The R^2^ and MSE values of the LMEs, the ANNs with LME random effects, and ANN with participant scalar embeddings are displayed on Table 4. For the ANN models, the 95% confidence intervals are calculated from 30 runs.

**Table 4:** Comparison of LME models and ANN Mixed Effects models using R^2^ for comparison.

**Model**	**Experiment**	**R^2^**	**MSE**
LME	Closeness-to-God experiment 3	0.945	2.803
ANN with LME random effects	Closeness-to-God experiment 3	0.937 ± 0.000	2.340 ± 0.000
ANN with participant scalar embeddings	Closeness-to-God experiment 3	0.924 ± 0.003	2.850 ± 0.104
LME	LAMBI-A experiment 4	0.894	0.341
ANN with LME random effects	LAMBI-A experiment 4	0.835 ± 0.000	0.341 ± 0.000
ANN with participant scalar embeddings	LAMBI-A experiment 4	0.804 ± 0.006	0.405 ± 0.012

Top: comparison for dependent variable closeness-to-God. Bottom: comparison for God concepts. Table 4 shows the comparative results of Linear Mixed Effects Models vs. an Artificial Neural Network Model by the metrics of R^2^ and Mean Squared Error.

#### 3.2.2 Study 2.1 summary

Study 2.1 demonstrates the similarities between an LME model and specific ANN architecture designed to mimic an LME. The results demonstrate that the models possess similar predictive power. In our implementation of the ANN with participant scalar embeddings, these embeddings act as random effects but are not constrained in the same way that random effects in the LME are. Our results demonstrate that removing this constraint from the fitting of the model does not give the ANN a unilateral advantage over the LME, and may make it more difficult for the model to fit optimally. By showing comparability of LMEs and this particularly simple ANN design, Study 2.1 establishes the credibility of ANNs as a tool for analyzing time-dependent data. Thus, we move to the more complex ANNs of Study 2.2 with well-earned confidence in the analytical method.

#### 3.2.3 Study 2.2 results

**Figure 2:**
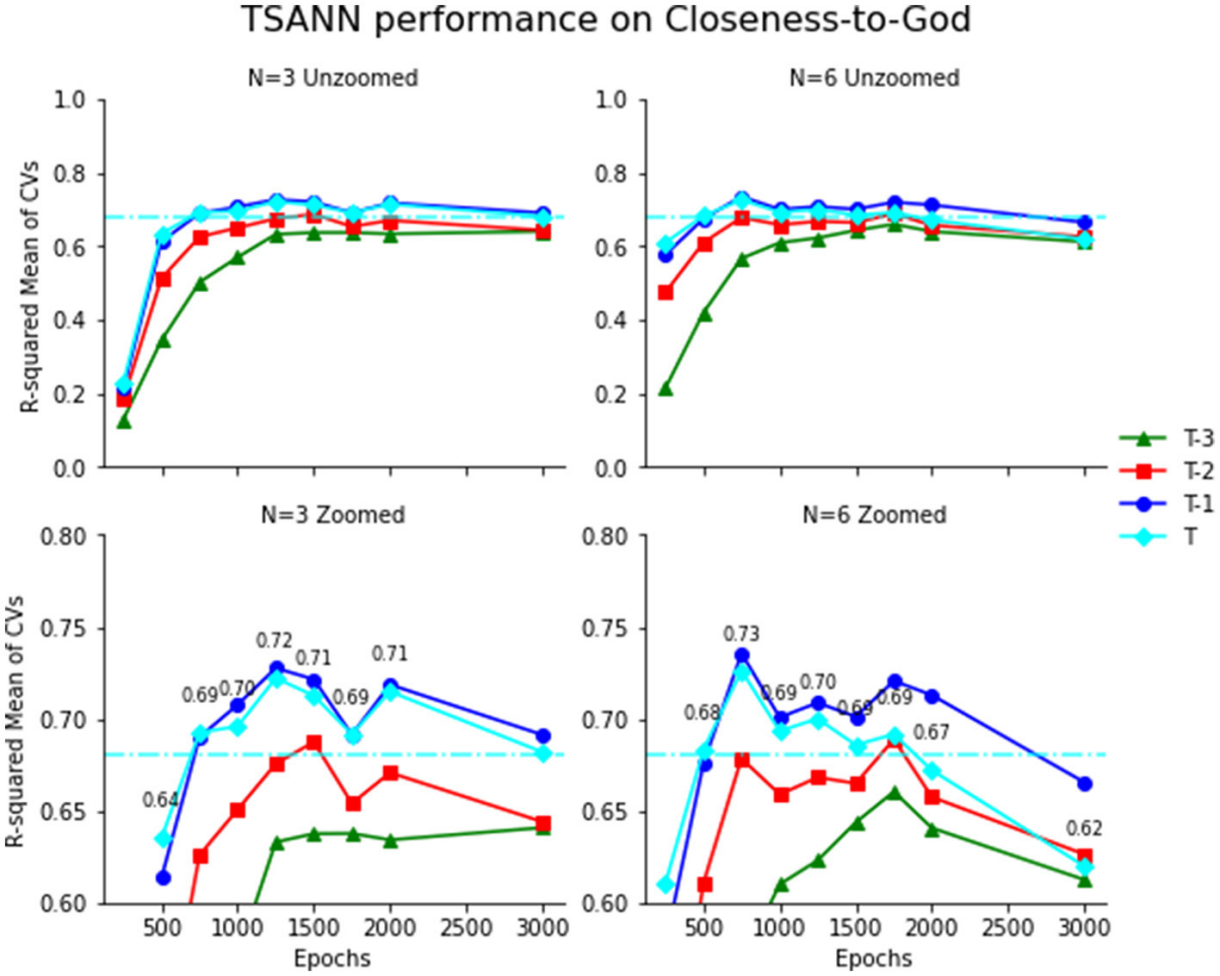
TSANN performance on closeness-to-God. Closeness-to-God (left) measures predicted by the participant-stratified 3-fold cross-validated Temporal ANN for times T (cyan), T-1 (blue), T-2 (red), and T-3 (green), and compared to the standard defined by the LME. LAMBI-Authoritarian God concept measures are on the right. The first row on both plots has a vertical scale from 0 to 1, and the second row repeats the same data with a vertical scale of [0.6, 0.8] and numerical labels of the time T (cyan) measurement.

After 30 repetitions, the participant-stratified 3-fold cross-validated LME regression model yielded a mean R^2^ of 0.679 (SD = 0.047) for closeness-to-God. This functions as the standard for evaluating results from the participant-stratified 3-fold cross-validated temporal series artificial neural network model (TSANN) in what follows. After running the hyperparameter variations on TSANN, we observed that several combinations of the number of hidden neurons per node group (N) and epochs (training periods of the model) could significantly outperform the LME model for predicting time T outcomes, in addition to also producing predictions for T-1 day, T-2 day, and T-3 day outcomes. In the closeness-to-God experiments, N values from 3 to 6 achieved their optimal performances over varying numbers of epochs or training runs. We have highlighted the *N* = 3 and *N* = 6 values across all epochs in Figure 2, with the second row of Figure 2 repeating the same plots with a zoomed-in y-axis of 0.6–0.8 and cyan text labels of the R-squared values for the time T outcome (Supplementary Table 9). The horizontal dotted line is the corresponding participant-stratified 3-Fold cross-validated value from the LME (0.679).

We chose to highlight *N* = 3 and *N* = 6 to show that while *N* = 6 is able to achieve higher R^2^ values, lower values of N still surpass the LME and can perform very similarly.

For the *N* = 6 epochs = 750 iterations of TSANN in the closeness-to-God condition, the mean and standard deviation was 0.726 and 0.040 over 30 replications. In a Welch's *t*-test comparison against the participant-stratified cross-validated LME (mean = 0.681, SD = 0.024), the performances of these models are statistically significantly different (*t* = 5.31, DoF = 47.51, *p* < 0.001; see Supplementary Table 10 for full outputs).

**Figure 3:**
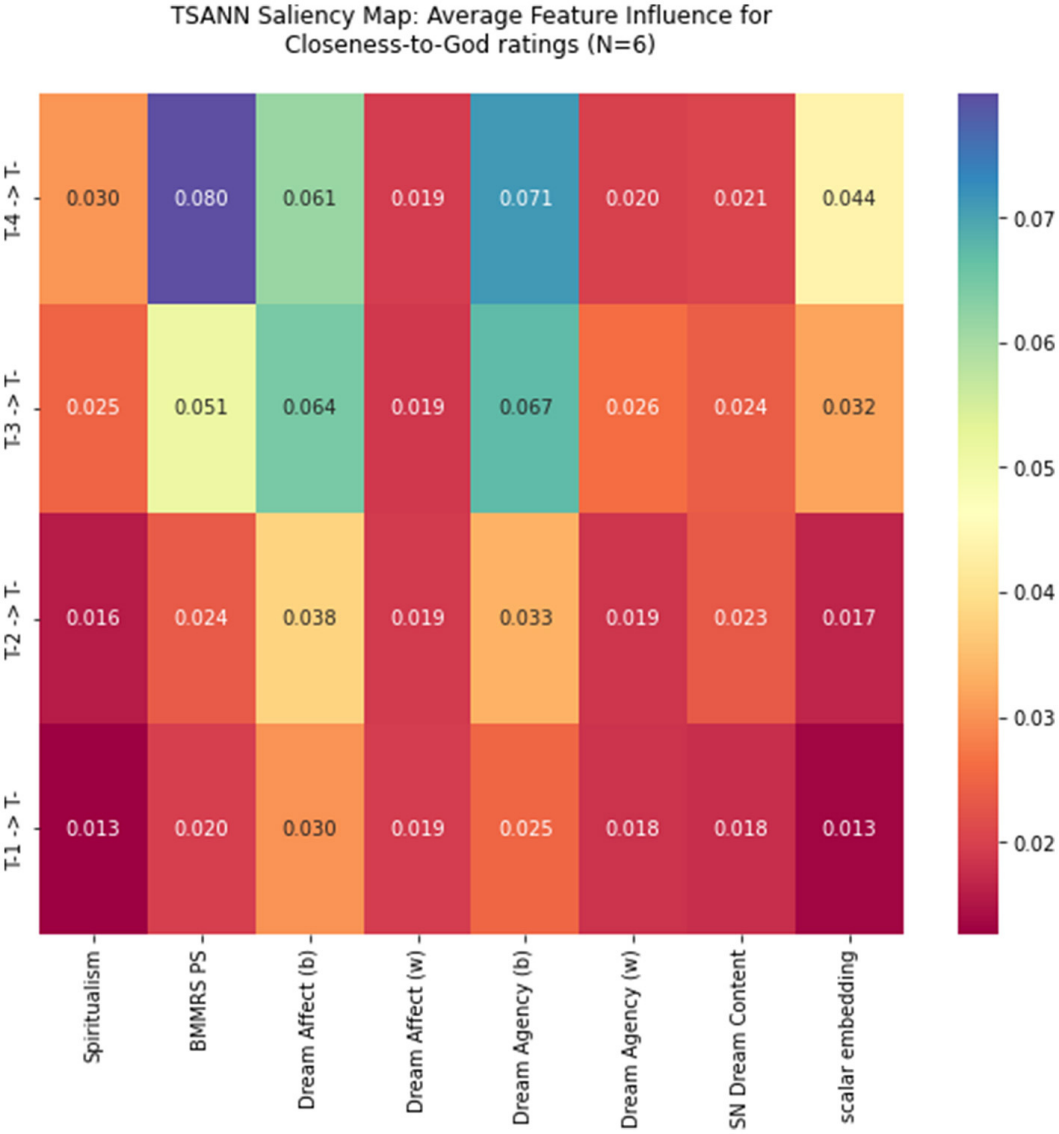
Saliency maps of mean feature influences and variances for fitting to the time T outputs in TSANN. The values in the saliency maps correspond to the gradients TSANN calculates for each input, which tell us how sensitive TSANN is to changes in these inputs. In the mean figures, the gradients are normalized such that they all sum to one, and the relative differences in the gradients for each input tell us the relative influence/importance that these inputs hold in the model. The variances tell us how much variation there was in these gradients across all runs.

Figure 3 displays the saliency maps from the *N* = 6 best epochs runs of TSANN. BMMRS Positive Spirituality and the between-subjects effects of dream affect and agency play the strongest roles in generating the time T predictions. Notably, the model placed a greater emphasis on the time T-3 and T-4 inputs than the T-2 and T-1 inputs. Also notably, the within-subjects values (w) for dream affect and agency tended to have a third to half the influence of the between-subjects values (b), which means the influence of the within-participants values are non-trivial, if lower in magnitude. Likewise, the supernatural dream content contributed a similar amount as the between-subjects values of dream affect and agency.

In the LAMBI-Authoritarian condition, the LME marginal R^2^ values were consistently less than or equal to zero, indicating that the model was unable to account for any variance when performing participant-stratified 3-Fold cross-validation. TSANN, on the other hand, did achieve positive R^2^ values over the hyperparameter variation experiments performed. However, high variances over experiment repetitions indicate TSANN was unable to generalize information in the data when being cross-validated. For instance, N = 2 epochs = 1,250 TSANN had a mean R^2^ value of 0.255 (SD 0.110) and was among the highest performing parameter variations. A standard deviation of nearly half the mean is in high contrast to the standard deviations in the closeness-to-God case. Due to this, it would not be appropriate to perform *post-hoc* saliency map analysis for the LAMBI-Authoritarian condition.

#### 3.2.4 Study 2.2 summary

Study 2.2 demonstrates how TSANN can model time T predictions to produce higher R^2^ values than the LME, and then breaks down the influence of the different features using saliency maps. These results give a much more fine-grained analysis of the effects of different inputs across the full 4-day time sequence discovered empirically in Study 1. However, these tests were only able to be fully performed in the closeness-to-God case. In the LAMBI-Authoritarian case, the LME was unable to account for variance when applying cross-validation, and TSANN was unable to generalize the information in the data. There can be many reasons why this was the case, whether it be insufficient amounts of data to properly measure the relationships in the data and account for noise, outliers in the data, or the model may be too sensitive to overfitting in this research application. However, given that the LME also could not generalize when applying cross-validation, and that both the LME and TSANN could generalize in the closeness-to-God case, we do not take TSANN's inability to generalize on the LAMBI-Authoritarian case to be a reason to discount our TSANN findings in the closeness-to-God case.

#### 3.2.5 Study 2.3 results

In Study 2.3 we trained modified versions of the temporal neural network model for different time lags, using different iterations of the lagged analysis (Supplementary Figures S3–S5). These analyses were only performed for the closeness-to-God condition, due to finding in study 2.2 that TSANN could not generalize in the LAMBI-Authoritarian case.

First, we determined the optimal number of hidden nodes (N) to evaluate performance across different time lags. We found that *N* = 6 was the N parameter that performed best or statistically equivalently to the best N for each of the day lags. For *N* = 6 on the closeness-to-God tests, the optimal epochs for the 4-day lag, 3-day lag, 2-day lag, and 1-day lag versions of TSANN were 500, 750, 1,000 and 1,000 epochs, respectively.

Shapiro-Wilk tests indicated non-normality within each group (*p* < 0.01 for all; specifically, *p* = 0.0083, 0.000, 0.000, 0.002). However, visual inspection of histograms revealed approximately bell-shaped distributions resembling normal curves, and Q-Q plots showed only minor deviations from normality. Given the computational nature of the data and the high number of cross-validation replications (*n* = 200), perfect normality was not expected. Levene's test indicated heterogeneity of variances across groups (*p* = 0.004). Based on these diagnostics, we proceeded with Welch's ANOVA and Games-Howell *post-hoc* analysis.

Welch's ANOVA revealed a statistically significant difference between groups (*p* < 0.001, partial eta-squared ηp^2^ = 0.0297). The Games-Howell *post-hoc* analysis showed that the 3-day and 4-day lagged models significantly outperformed the 1-day lagged model (*p* = 0.031 and *p* = 0.000, respectively). Additionally, the 4-day lagged model significantly outperformed the 2-day lagged model (*p* = 0.003). No other comparisons were statistically significant, indicating no detectable differences between the 1-day and 2-day lagged models, or between the 3-day and 4-day lagged models. Table 5 shows the results of a Games-Howell analysis, indicating which pairs of means are significantly different.

**Table 5:** ANOVAs showed significantly different means in the different time lagged models' performances.

**Outcome**		**Closeness-to-God**	
**Epochs**		**Optimal**	
**Lags compared**		Δμ	**p-adj**
1	2	0.007	0.699
1	3	0.015	0.031
1	4	0.025	0.000
2	3	0.009	0.397
2	4	0.019	0.003
3	4	−0.010	0.167

This Games-Howell analysis investigates which mean comparisons contributed.

## 4 Discussion

In this set of studies, we examined the cognitive dynamics of supernatural content dreams across a 2 week period and waking life spirituality ratings and God images. In Study 1, we used computational linguistic tools to analyze supernatural content in a large dataset, which we built upon in study 1.1 by looking at participant ratings of supernatural content across a 2-week period in an intensive longitudinal design collecting data in the home using previously validated survey instruments, diaries and the Dreem 3 Headband. In study 1.2 we then investigated the impacts of dream content and sleep architecture on waking God-concepts and images. In Study 2.1 we used a simple, one-perceptron ANN with no activation function to mimic the mixed effects LMEs to assess the similarities and differences between LMEs and ANNs. In Study 2.2 we used a TSANN to evaluate the theory explaining the association of study 1.2, obtaining computational support for the causal explanation that dream content *causes* changes in day-to-day experiences of closeness to God and God concepts, with finer granularity regarding temporal effects than the LMEs in study 1.2. The saliency maps of TSANN in study 2.2 showed that T-4 and T-3 effects predictors influenced the model outputs more than the T-1 and T-2 inputs, demonstrating the importance of the time-lagged effects in daily closeness-to-God ratings. Findings from study 2.3 further demonstrate this relationship, in showing that the 1-day and 2-day lagged TSANN models were not as effective as the 3-day and 4-day time lagged models, although there were not statistically significant differences between the 3-day and 4-day lagged models.

We tested two major hypotheses on dream supernatural content—namely that 1) supernatural dream content is reliably associated with daytime spirituality and god concepts and that 2) dream content features (including dreamer agency and SA-related content) is significantly associated with daily R/S experiences.

We tested the characteristics of supernatural dream content based on participant ratings and external ratings of dreamer agency in Study 1.1. This analysis revealed that supernatural dreams have lower levels of dream agency, and are rated as more bizarre, strange, and scary. We then assessed the impact of dream content variables and sleep architecture on two dimensions of God-images (closeness and authoritarianism) in study 1.2. These studies demonstrated a significant effect of these dream content features on these SA dimensions with both concurrent and lagged effects, as well as a positive relationship between REM% and both dimensions of daily God rating outcomes.

In studies 2.2 and 2.3, we constructed a deep temporal neural network, TSANN, which we trained on data gathered in study 1. We used participant-stratified K-fold cross-validation procedures and ran repeated training replications on study 1 data, which revealed optimal values for closeness-to-God ratings after 500 to 1,250 learning epochs and precisely reproduced the 4-day lagged effects discovered in study 1. These results, along with those from study 1, strongly suggest that lower agency in the dream and dream affect are causally influencing daily closeness-to-God, but were inconclusive for LAMBI-Authoritarian outcomes.

Thus, across both studies in this project, lower dreamer agency and negative dream affect both characterized dreams with supernatural content and impacted daily changes in closeness-to-God. These results therefore support our hypotheses that dream content is significantly associated with daytime spirituality and religious cognition, and are consistent with the McNamara et al. (2018) and Nordin (2024a,b) descriptive studies summarized in the Introduction. We suggest that the same cognitive processes (mentalizing, social simulation, relational thinking) that enable individuals to relate to SAs during the day are calibrated within and updated during dreams. Lower dream agency occurring in REM dreams sets up the cognitive conditions necessary for the dreaming mind/brain to simulate other dream characters who are invested with supernormal levels of agency and supernatural powers. We note that it is not possible from this study to assess whether or not levels of low dreamer agency cause higher levels of supernatural content in dreams or whether the occurrence of supernatural content lowers dreamer agency. We contend only that our results and previous literature suggest a strong link between these two variables and further bi-directional studies are needed to tease out this relationship further.

It is important to note, however, that within the mixed effects models in Study 1 overt supernatural content in a dream was not predictive of next-day relational or conceptual God-image ratings, but only operated at a 4-day lag, and only impacted the closeness-to-God dimension. This suggests that effects of reduced dreamer agency take some amount of time to become operative in the cognitive system. Once operative, these dream-dependent mechanisms start to influence daily closeness-to-God and God image ratings. That lag appeared to be approximately 4 days, which we confirmed through the construction of the Temporal Neural Net model. We suggest that the temporal lag effect documented here is broadly consistent with the now relatively abundant evidence (see Wagner et al., 2001) which demonstrates that the REM sleep and dream system functions as an emotional memory processing system. Our testing of different levels of the lags using the temporal ANN indicates that there is at least a 2–3 day lag in the consolidation of dream events into both relational and conceptual schemas of God-images. This finding points to the need for future work to consider dream effects beyond the next morning.

We contend that this lagged process means individuals are attempting to integrate associated emotional memories/fear memories and update their social schemas over several nights via sleep and dreaming. Thus, dream content influences God-images over several nights because they reflect integration attempts, and the greater the number of attempts, the more likely integration occurs. While dream affect had immediate and lagged effects on next-day closeness-to-God and authoritarian God image, agency and supernatural content only influenced closeness-to-God after several days had passed. This suggests that while overt emotionality influences God-images immediately, conceptual aspects of dream content take more time to become consolidated into memory and to update the social schemas behind representations of God. The negative impact of dream agency and supernatural content on closeness-to-God thus may indicate that simulating SAs in dreams may be in tension with waking relational schemas of religious SAs, like God, when these dreams are negative or low agency.

How might individual differences in belief influence the impacts of dream content on God-images more generally? In study 1, Baseline BMMRS positive spirituality was a powerful positive predictor of closeness-to-God, indicating that this factor should be conceptualized as largely corresponding to positively appraised personal theism. Similarly, traditional religious beliefs, which ask individuals about their belief in hell and the devil, were strongly associated with longitudinal concepts of an authoritarian God, which indicates that authoritarian God-image is highly tied to the ability to punish as well as the existence of negative oppositional SAs like the devil. We also found that individual belief on the “spiritualism” subscale of the RPBS, which primarily deals with questions about the independence of the soul or spirit from the body, predicted higher levels of daily closeness-to-God ratings. Higher levels of felt closeness-to-God corresponding with endorsing this dualistic view of the soul is likely due to the necessity of conceptualizing God as a mind or soul without a body in order to cultivate a felt sense of closeness to an intangible agent. This finding tracks with a wide literature in the cognitive science of religion postulating that mind-body dualism is a key ingredient in SA cognitions (see Gut et al., 2021). It is interesting that spiritualism also had strong interaction effects for both dream agency and dream affect. The finding that dream agency has a significant effect on closeness to god in interaction with spiritualism but does not have an effect on its own indicates that mind-body dualist beliefs may be an important facilitator for the impact of dream experiences on ongoing relational experiences of God. The negative relationship for the interaction of dream affect and spiritualism on closeness-to-God compared to both positive main effects is more puzzling, however. We speculate that for individuals with higher levels of spiritualism, dreams are more likely to be appraised as actual visitations or messages from an outside force. Highly negative dreams for these individuals may thus trigger increased closeness-to-God as they seek protection against the negative emotional traces left over from their dreams.

We have seen in our studies that varying content in dreaming was significantly associated with changes in daytime feelings of closeness-to-God as well as daytime changes in basic conceptions of God. But how might these subjective experiences then go on to affect other daytime social behaviors? As we mentioned in the Introduction, there is strong evidence that one's basic God image influences a wide array of outcomes and behaviors including health, anxiety levels, general wellbeing, willingness to cheat, levels of social trust and political attitudes, among others.

## 5 Conclusions

We tested 2 major hypotheses on –dream content and daytime spirituality/religious cognition, namely that 1) there are significant associations between dream content and daytime spirituality and that 2) selected dream content features (e.g., dreamer agency) are significantly associated with wakingdaily religious or spiritual experiences.

Supernatural content occurs spontaneously and commonly in dreams. Supernatural content in dreams is associated with reduction in dreamer agency and experiences of bizarreness. Dreamer agency and felt affect during the dream also influences waking God-images in both relational and conceptual dimensions even when accounting for baseline differences in religiosity and spiritual beliefs. Supernatural content in dreaming only influences conceptual God-images with a 4-day lag, indicating that dreamer agency may serve as a key mediator between supernatural experiences in dreaming and changes in waking religious beliefs and attitudes. We suggest that these results indicate that causally effective SA concepts are generated in REM dreaming and that dreams regulate key elements of daily levels of spirituality.

There are several limitations of this study. Our methods for training our classifiers to identify supernatural dreams relied on a general reference to supernatural content, and a more fine-grained content analysis may have yielded different results. While inspection of our classifiers found them to be broadly accurate in identifying supernatural content occurrence in the longitudinal dataset, machine learning classifiers of these kinds may be subject to false positives or negatives, with a trade-off of providing higher levels of replicability and consistency.

Our longitudinal sample showed an unusually high level of negative affect dreams frequency compared to the overall population. This is likely due to our recruiting materials, some of which specifically targeted individuals with frequent nightmares, but may have led to a self-selection bias. Furthermore, our use of mixed-effects models means that our findings are based on overall trends within the sample, but there may always be hidden, or unknown factors not being accounted for that can influence the hypothesized relationships. In addition, our lags were constructed using days as the primary unit of time, while the temporal dynamics of emotional and conceptual shifts likely unfold in a more ongoing and non-linear fashion. Future work would benefit from employing techniques like event sampling and dynamic modeling that incorporates time to gain a more fine-grained analysis of these temporal shifts.

Black-box algorithms for sleep architectural scoring have inherent limitations. For example, the DH automatic sleep scoring algorithm only reaches about 55% accuracy when detecting N1 sleep, which could affect accuracy of the sleep stage classifications and distributions (Arnal et al., 2020a,b). Since participants were responsible for the placement of the headband, the signal quality varied throughout their 2-week participation in the study. We instructed participants to start the recording when they were ready to start trying to fall asleep and end the recording when they woke up, but it is possible that some did not follow this instruction exactly, resulting in extra wake time being recorded, which would affect related metrics. It should also be noted that since the headband requires a smartphone or tablet along with strong, reliable Wi-Fi, those without full access to these resources were not able to participate, potentially biasing the sample.

Study 2, particularly Studies 2.2 and 2.3, are limited by low amounts of data. The data processing necessary for TSANN reduced sample counts to 268 entries over 41 participants and 311 entries over 47 participants for the closeness-to-God and LAMBI-Authoritarian conditions. ANNs often require more data samples than regression models to produce meaningful results. While TSANN was able to produce meaningful results with our data set in the case of closeness-to-God, reproducing these results across more studies may be necessary. TSANN and the LME could not generalize in the LAMBI-Authoritarian case, and this is likely at least in part due to data limitations. Another limitation of Study 2 is the inherent black-box nature of ANNs, which we aimed to mitigate through using *post-hoc* saliency maps analysis to “look inside” TSANN and overcome much of this limitation. Another useful method of analysis to “look inside” ANNs is called ablation testing, where pieces of the model are removed, or ablated, before model evaluation, offering insight into the importance of the ablated piece of the model. In future work, we aim to study TSANN by ablating different inputs of the model and temporal connections.

## Data Availability

The raw data supporting the conclusions of this article will be made available by the authors, without undue reservation.

## References

[B1] Anjum M. F. Smyth C. Zuzuárregui R. Dijk D. J. Starr P. A. Denison T.. (2024). Multi-night cortico-basal recordings reveal mechanisms of NREM slow-wave suppression and spontaneous awakenings in Parkinson's disease. Nat. Commun. 15:1793. 10.1038/s41467-024-46002-7 38413587 PMC10899224

[B2] Arnal P. J. Thorey V. Debellemaniere E. Ballard M. E. Hernandez A. B. Guillot A.. (2020a). The dreem headband compared to polysomnography for electroencephalographic signal acquisition and sleep staging. Sleep 43:zsaa097. 10.1093/sleep/zsaa097 32433768 PMC7751170

[B3] Arnal P. J. Thorey V. Debellemaniere E. Mordret E. Llamosi A. Chouraki A. (2020b). Sleep science at home: delivering sleep assessment and digital CBT-I at scale. Sleep 43:A209. 10.1093/sleep/zsaa056.543

[B4] Asp A. Lund F. Benedict C. Wasling P. (2022). Impaired procedural memory in narcolepsy type 1. Acta Neurol. Scand. 146, 186–193. 10.1111/ane.13651 35652281 PMC9544773

[B5] Beacon (2024). Dreem headband. Beacon Biosignals. Available online at: https://beacon.bio/dreem-headband/ (Accessed August 1, 2024).

[B6] Birch I. C. (2021). Using wearable EEG to quantify associations between sleep architecture, anxiety, and fear memory (PhD thesis). Cardiff University. Available online at: https://orca.cardiff.ac.uk/id/eprint/145872/

[B7] Bulkeley K. (n.d.). Sleep and Dream Database. Available online at: https://sleepanddreamdatabase.org/ (Accessed August 1, 2024).

[B8] Caruana R. (1997). Multitask learning. Mach. Learn. 28, 41–75. 10.1023/A:1007379606734

[B9] Chinoy E. D. Cuellar J. A. Jameson J. T. Markwald R. R. (2022). Performance of four commercial wearable sleep-tracking devices tested under unrestricted conditions at home in healthy young adults. Nat. Sci. Sleep 14, 493–516. 10.2147/NSS.S348795 35345630 PMC8957400

[B10] Davis E. B. Granqvist P. Sharp C. (2021). Theistic relational spirituality: development, dynamics, health, and transformation. Psychol. Relig. Spirit. 13:401. 10.1037/rel0000219

[B11] Davis E. B. Moriarty G. L. Mauch J. C. (2013). God images and god concepts: definitions, development, and dynamics. Psychol. Relig. Spirit. 5:51. 10.1037/a0029289

[B12] Dezutter J. Luyckx K. Schaap-Jonker H. Büssing A. Corveleyn J. Hutsebaut D. (2010). God image and happiness in chronic pain patients: the mediating role of disease interpretation. Pain Med. 11, 765–773. 10.1111/j.1526-4637.2010.00827.x 20353410

[B13] Domhoff G. W. Schneider A. (2018). Are dreams social simulations? Or are they enactments of conceptions and personal concerns? An empirical and theoretical comparison of two dream theories. Dreaming 28, 1–23. 10.1037/drm0000080

[B14] Domhoff G. W. Schneider A. (n.d.). DreamBank. Available online at: https://dreambank.net/

[B15] Fetzer Institute (1999). Multidimensional measurement of religiousness/spirituality for use in health research: a report of the Fetzer Institute/National Institute on Aging Working Group. Available online at: https://backend.fetzer.org/sites/default/files/resources/attachment/%5Bcurrent-date%3Atiny%5D/Multidimensional_Measurement_of_Religousness_Spirituality.pdf (Accessed August 1, 2024).

[B16] Fogli A. Aiello L. M. Quercia D. (2020). Our dreams, our selves: automatic analysis of dream reports. R. Soc. Open Sci.7:192080. 10.1098/rsos.192080 32968499 PMC7481704

[B17] Fox M. D. Snyder A. Z. Vincent J. L. Corbetta M. Van Essen D. C. Raichle M. E. (2005). The human brain is intrinsically organized into dynamic, anticorrelated functional networks. Proc. Natl. Acad. Sci. U.S.A. 102, 9673–9678. 10.1073/pnas.0504136102 15976020 PMC1157105

[B18] Froese P. Bader C. (2008). Unraveling religious worldviews: the relationship between images of God and political ideology in a cross-cultural analysis. Sociol. Q.49, 689–718. 10.1111/j.1533-8525.2008.00132.x

[B19] Garten J. Hoover J. Johnson K. M. Boghrati R. Iskiwitch C. Dehghani M. (2018). Dictionaries and distributions: combining expert knowledge and large scale textual data content analysis. Behav. Res. Methods 50, 344–361. 10.3758/s13428-017-0875-9 28364281

[B20] González D. A. Wang D. Pollet E. Velarde A. Horn S. Coss P.. (2024). Performance of the dreem 2 EEG headband, relative to polysomnography, for assessing sleep in Parkinson's disease. Sleep Health 10, 24–30. 10.1016/j.sleh.2023.11.012 38151377

[B21] Grand G. Blank I. A. Pereira F. Fedorenko E. (2022). Semantic projection recovers rich human knowledge of multiple object features from word embeddings. Nat. Hum. Behav. 6, 975–987. 10.1038/s41562-022-01316-8 35422527 PMC10349641

[B22] Grootendorst M. (2022). BERTopic: Neural topic modeling with a class-based TF-IDF procedure. arXiv [Preprint]. arXiv:2203.05794.

[B23] Gut A. Lambert A. Gorbaniuk O. Mirski R. (2021). Folk beliefs about soul and mind: cross-cultural comparison of folk intuitions about the ontology of the person. J. Cogn. Cult. 21, 346–369. 10.1163/15685373-12340116

[B24] Hardy A. (1981). The Spiritual Nature of Man: A Study of Contemporary Religious Experience. Tijdschrift voor Filosofie, 43.

[B25] Hobson J. A. Pace-Schott E. F. Stickgold R. (2000). Dreaming and the brain: toward a cognitive neuroscience of conscious states. Behav. Brain Sci. 23, 793–842. 10.1017/S0140525X00003976 11515143

[B26] Holzinger B. Mayer L. Barros I. Nierwetberg F. Klösch G. (2020). The dreamland: validation of a structured dream diary. Front. Psychol. 11:585702. 10.3389/fpsyg.2020.585702 33178086 PMC7596900

[B27] Honnibal M. Montani I. (2017). spaCy 2: Natural language understanding with Bloom embeddings, convolutional neural networks and incremental parsing. Available online at: https://spacy.io/ (Accessed August 1, 2024).

[B28] Hornik K. Stinchcombe M. White H. (1989). Multilayer feedforward networks are universal approximators. Neural Netw. 2, 359–366. 10.1016/0893-6080(89)90020-8

[B29] Johnson D. P. (2016). God is Watching You: How the Fear of God Makes us Human. New York, NY: Oxford University Press.

[B30] Johnson K. A. Okun M. A. Cohen A. B. Sharp C. A. Hook J. N. (2019). Development and validation of the five-factor LAMBI measure of god representations. Psychol. Relig. Spirit. 11, 339–349. 10.1037/rel0000207

[B31] Johnstone B. Yoon D. P. (2009). Relationships between the brief multidimensional measure of religiousness/spirituality and health outcomes for a heterogeneous rehabilitation population. Rehabil. Psychol. 54, 422–431. 10.1037/a0017758 19929124

[B32] Kozlowski A. C. Taddy M. Evans J. A. (2019). The geometry of culture: analyzing the meanings of class through word embeddings. Am. Sociol. Rev. 84, 905–949. 10.1177/0003122419877135

[B33] Llewellyn S. (2013). Such stuff as dreams are made on? Elaborative encoding, the ancient art of memory, and the hippocampus. Behav. Brain Sci. 36, 589–607. 10.1017/S0140525X12003135 24304746

[B34] Lynn C. D. Schell L. M. (2024). Why religion and spirituality are important in human biological research. Am. J. Hum. Biol. 36:e24106. 10.1002/ajhb.24106 38767192

[B35] Macrae C. N. Moran J. M. Heatherton T. F. Banfield J. F. Kelley W. M. (2004). Medial prefrontal activity predicts memory for self. Cereb. Cortex 14, 647–654. 10.1093/cercor/bhh025 15084488

[B36] Mageo J. M. Sheriff R. E. eds. (2021). New Directions in the Anthropology of Dreaming. Routledge. 10.4324/9781003037330

[B37] Mahmoud A. Mohammed A. (2024). Leveraging hybrid deep learning models for enhanced multivariate time series forecasting. Neural Process. Lett. 56, 1–25. 10.1007/s11063-024-11656-3

[B38] Maquet P. Peters J. Aerts J. Delfiore G. Degueldre C. Luxen A.. (1996). Functional neuroanatomy of human rapid-eye-movement sleep and dreaming. Nature 383, 163–166. 10.1038/383163a0 8774879

[B39] Maria L. M. Quercia D. Foli A. (2020). Our Dreams, Our Selves: Automatic Interpretation of Dream Reports. Dryad Digital Repository.

[B40] McAdams D. P. (2001). Coding Autobiographical Episodes for Themes of Agency and Communion (Unpublished manuscript). Northwestern University, Evanston, IL.

[B41] McNamara P. (2016). Dreams and Visions: How Religious Ideas Emerge in Sleep and Dreams. Santa Barbara, CA: Praeger. 10.5040/9798400642470

[B42] McNamara P. (2022). The Cognitive Neuroscience of Religious Experience: Decentering and the Self. New York, NY: Cambridge University Press. 10.1017/9781108973496

[B43] McNamara P. Bulkeley K. (2015). “Dreams as a source of religious experience,” in Science and the World's Religions (Vol. 2), eds. P. McNamara and W. J. Wildman (Praeger), 109–128.

[B44] McNamara P. Johnson P. McLaren D. Harris E. Beauharnais C. Auerbach S. (2010). REM and NREM sleep mentation. Int. Rev. Neurobiol. 92, 69–86. 10.1016/S0074-7742(10)92004-7 20870063

[B45] McNamara P. Teed B. Pae V. Sebastian A. Chukwumerije C. (2018). Supernatural agent cognitions in dreams. J. Cogn. Cult. 18, 428–450. 10.1163/15685373-12340038

[B46] Mencken F. C. Bader C. D. Embry E. (2009). In god we trust: images of god and trust in the United States among the highly religious. Sociol. Perspect. 52, 23–38. 10.1525/sop.2009.52.1.23

[B47] Mikolov T. Chen K. Corrado G. Dean J. (2013). Efficient estimation of word representations in vector space. arXiv [Preprint]. arXiv:1301.3781. 10.48550/arXiv.1301.3781

[B48] Muurling M. De Boer C. Kozak R. Religa D. Koychev I. Verheij H.. (2021). Remote monitoring technologies in Alzheimer's disease: design of the RADAR-AD study. Alzheimers Res. Ther. 13:89. 10.1186/s13195-021-00825-4 33892789 PMC8063580

[B49] Nordin A. (2024a). Gauging oneiromancy—the cognition of dream content and cultural transmission of (supernatural) divination. Relig. Brain Behav. 14, 161–182. 10.1080/2153599X.2023.2172068

[B50] Nordin A. (2024b). Imagery of ritual actions in religious dreaming: steps toward a theory and method of the cognition of dreamt ritual interaction. Religion 54, 271–296. 10.1080/0048721X.2023.2284200

[B51] Northoff G. Bermpohl F. (2004). Cortical midline structures and the self. Trends Cogn Sci. 8, 102–107. 10.1016/j.tics.2004.01.004 15301749

[B52] Paquette A. (2018). The interpretation of independent agents and spiritual content in dreams. Int. J. Dream Res. 11, 86–105. 10.11588/ijodr.2018.2.41217

[B53] Pennycook G. Cheyne J. A. Seli P. Koehler D. J. Fugelsang J. A. (2012). Analytic cognitive style predicts religious and paranormal belief. Cognition 123, 335–346. 10.1016/j.cognition.2012.03.003 22481051

[B54] Pépin J. Bailly S. Mignot E. Gaucher J. Chouraki A. Cals-Maurette M.. (2022). Digital markers of sleep architecture to characterize the impact of different lockdown regimens on sleep health during the COVID-19 pandemic. Sleep 45:zsac074. 10.1093/sleep/zsac074 35429392 PMC9189935

[B55] Rehurek R. Sojka P. (2011). Gensim-python framework for vector space modelling. NLP Centre, Faculty of Informatics, Masaryk University, Brno, Czech Republic, 3:2.

[B56] Rittenhouse C. D. Stickgold R. Hobson J. A. (1994). Constraint on the transformation of characters, objects, and settings in dream reports. Conscious. Cognit. 3, 100–113. 10.1006/ccog.1994.1007

[B57] Rodriguez P. L. Spirling A. (2021). Word embeddings: what works, what doesn't, and how to tell the difference for applied research. J. Polit. 84:101–115. 10.1086/715162

[B58] Saguin E. Gomez-Merino D. Sauvet F. Leger D. Chennaoui M. (2021). Sleep and PTSD in the military forces: a reciprocal relationship and a psychiatric approach. Brain Sci. 11:1310. 10.3390/brainsci11101310 34679375 PMC8533994

[B59] Schredl M. Mönch J. H. (2023). Dreaming of god and the role of faith in everyday life: an empirical study. Pastor. Psychol. 72, 469–478. 10.1007/s11089-023-01083-x

[B60] Schwartz S. Clerget A. Perogamvros L. (2022). Enhancing imagery rehearsal therapy for nightmares with targeted memory reactivation. Curr. Biol. 32, 4808–4816.e4. 10.1016/j.cub.2022.09.032 36306786

[B61] Sharp C. A. Johnson K. A. (2020). Assessing spirituality on two dimensions: closeness to god and focal orientation. Int. J. Psychol. Relig. 30, 48–67. 10.1080/10508619.2019.1633853

[B62] Simonyan K. Vedaldi A. Zisserman A. (2013). Deep inside convolutional networks: visualising image classification models and saliency maps. arXiv [Preprint]. arXiv:1312.6034. 10.48550/arXiv.1312.6034

[B63] Song K. Tan X. Qin T. Lu J. Liu T. Y. (2020). Mpnet: Masked and permuted pre-training for language understanding. Adv. Neural Inf. Process. Syst. 33, 16857–16867.

[B64] Sosis R. Wildman W. J. Bulbulia J. Schjoedt U. (2017). Hilbert problems in the scientific study of religion. Relig. Brain Behav. 7, 277–278. 10.1080/2153599X.2017.1385202

[B65] Speer R. Chin J. Havasi C. (2017). ConceptNet 5.5: an open multilingual graph of general knowledge. Proc. AAAI Conf. Artif. Intell. 31, 4444–4451. 10.1609/aaai.v31i1.11164

[B66] Stickgold R. Walker M. P. (2007). Sleep-dependent memory consolidation and reconsolidation. Sleep Med. 8, 331–343. 10.1016/j.sleep.2007.03.011 17470412 PMC2680680

[B67] Tausczik Y. R. Pennebaker J. W. (2010). The psychological meaning of words: LIWC and computerized text analysis methods. J. Lang. Soc. Psychol. 29, 24–54. 10.1177/0261927X09351676

[B68] Thorey V. Guillot A. Kanbi K. E. Harris M. Arnal P. J. (2020). 1211 assessing the accuracy of a Dry-EEG headband for measuring brain activity, heart rate, breathing and automatic sleep staging. Sleep 43:A463. 10.1093/sleep/zsaa056.1205

[B69] Thorey V. Harris M. Guillot A. Hernandez A. Arnal P. (2019). The dreem2 headband as an alternative to polysomnography for EEG signal acquisition, breathing and heart rate monitoring and sleep staging in healthy subjects. Sleep Med. 64:S383. 10.1016/j.sleep.2019.11.1068

[B70] Tobacyk J. J. (2000). Revised Paranormal Belief Scale (RPBS) [Database record]. APA PsycTests. Available online at: https://www.google.com/search?q= 10.1037/t14015-000

[B71] Tuominen J. Olkoniemi H. Revonsuo A. Valli K. (2022). ‘No man is an island': effects of social seclusion on social dream content and REM sleep. Br. J. Psychol. 113, 84–104. 10.1111/bjop.12515 34107065

[B72] Tuominen J. Stenberg T. Revonsuo A. Valli K. (2019). Social contents in dreams: an empirical test of the social simulation theory. Conscious. Cogn. 69, 133–145. 10.1016/j.concog.2019.01.017 30769273

[B73] Van Elk M. Aleman A. (2017). Brain mechanisms in religion and spirituality: an integrative predictive processing framework. Neurosci. Biobehav. Rev. 73, 359–378. 10.1016/j.neubiorev.2016.12.031 28041787

[B74] VanderWeele T. J. (2017). Religion and health: a synthesis. Spirit. Relig. Cult. Med. 419, 357–401. 10.1093/med/9780190272432.003.0022

[B75] Waeber A. Arnal P. J. Lecciso G. Albir D. Mignot E. Heinzer R. (2021). Acoustic stimulation time-locked to the beginning of sleep apnea events reduces oxygen desaturations: a pilot-study. Sleep Med. 78, 38–42. 10.1016/j.sleep.2020.12.006 33383395

[B76] Wagner U. Gais S. Born J. (2001). Emotional memory formation is enhanced across sleep intervals with high amounts of rapid eye movement sleep. Learn. Mem. 8, 112–119. 10.1101/lm.36801 11274257 PMC311359

[B77] West B. T. Welch K. B. Galecki A. T. (2022). Linear Mixed Models: A Practical Guide Using Statistical Software. New York, NY: Chapman and Hall/CRC. 10.1201/9781003181064

[B78] Wilt J. A. Exline J. J. (2022). Receiving a gift from god in times of trouble: links between gratitude to god, the affective circumplex, and perceived closeness to god. Ment. Health Relig. Cult. 25, 362–379. 10.1080/13674676.2022.2033710

[B79] Wolf T. Debut L. Sanh V. Chaumond J. Delangue C. Moi A.. (2020). “Transformers: state-of-the-art natural language processing,” in Proceedings of the 2020 Conference on Empirical Methods in Natural Language Processing: System Demonstrations, eds. Q. Liu, and D. Schlangen (Association for Computational Linguistics), 38–45. 10.18653/v1/2020.emnlp-demos.6

[B80] Zhang Y. Yang Q. (2021). A survey on multi-task learning. IEEE Trans. Knowl. Data Eng. 34, 5586–5609. 10.1109/TKDE.2021.3070203 PMC10619966 37915376

