# Supplementary material for: Dream content influences daily spirituality
Source: Front Psychol. 2025 Jul 28;16:1575174. doi: 10.3389/fpsyg.2025.1575174 (PMC12337890; doi:10.3389/fpsyg.2025.1575174)
Supplement: Supplementary file 1 [file Supplementary_file_1.docx]

***Supplementary Material***

# Supplementary Data

**Supernatural Classifier from Large Text Databases**

Before testing our longitudinal sample, we first sought to examine the frequencies and phenomenological features of the appearance of overt supernatural content such as angels, demons, fairies, etc. in dreams. To that end we selected a total of N = 39,095 dream reports archived at the Sleep and Dream Database (Bulkeley, n.d.), and the Dreambank database (Domhoff, 2018). These narratives were also compiled in Fogli et al. (Fogli et al., 2020), who condensed them into a dataset of reports of at least 50 words written in English.

The dream databases contain dream reports collected as part of research efforts from the 1960s to the present and are still available at the time of publication (Fogli et al., 2020). The Dreambank database (Domhoff & Scneider, n.d.) contains 38,000 reports in English ranging from 1960 to 2015, including individual dream diaries as well as cohort studies. The Sleep and Dream Database has been more recently developed by Kelly Bulkeley (Bulkeley, n.d.) and his associates, and it contains reports in English across a wider range of cultural and temporal settings, including dream journals from historical figures and dream narratives collected focused on specific topics, such as COVID-19 anxieties or dreams about political figures. We chose to cast a wide net to get a maximally diverse set of dream narratives. We excluded narratives with less than 50 words to get documents of sufficient complexity for scoring, yielding an N of 35,008 dream narratives total.

As noted above we provisionally defined “supernatural” content as any textual content dealing with an entity with unusual or supernormal powers or environments, or events outside of the range of natural causality. Since we are not targeting any specific religious tradition or culture, we cast a wide net to get a general sense of the semantic dimensions carried along with supernatural content.

For our analysis of the text, we utilized distributed dictionary approach utilizing word embeddings to capture a more nuanced range of semantic interrelations than traditional word count approaches such as Linguistic Inquiry and Word Count (LIWC) (Tausczik & Pennebaker, 2010), which calculate the co-occurrence of topics due to counting keywords in a domain-based lexicon (see the discussion in Garten et al., 2018). In the distributed dictionary approach, word vectors can also be averaged to generate a new vector that approximates the semantic space in between them. This enables two relevant procedures. First, we can search documents for similar terms to any search terms after filtering out stop words. Second, as described in Garten et al. (Garten et al., 2018), search domains can be further specified by the creation of averaged vectors of dictionaries of relevant concepts, making them into distributed dictionaries for semantic comparison. In addition, antonym pairs can be used to create vectors that represent a specific dimension between terms that otherwise might be represented as very close together in the semantic space, such as “good/bad” or “powerful/weak” (Kozlowski et al., 2019). A recent study using this type of semantic projection found that word embeddings contain a wealth of implicit knowledge, with ratings of the model matching human judgements across a variety of object categories and dimensions (Grand et al., 2022).

In constructing our distributed dictionaries for supernatural content, we first explored all synonyms for a variety of starting supernatural agent terms. We then iteratively evaluated and built dictionaries using different seed words or combinations of seed words. These were then clustered and mapped using Uniform Manifold Approximation (UMAP) and Hierarchical Density-Based Spatial Clustering of Applications with Noise (HDBSCAN) (Grootendorst, 2022) to create separate dictionaries that handled different “senses” of semantic areas of interest or to filter out noise words that might be retrieved by the supernatural dictionaries (supplementary figure 1). For example, a dictionary referencing “heaven” may also pull terms related to the sky unless those words are captured by a more generic dictionary for sky related terms (see the Section 2 of Supplementary Materials for the full process). Text processing and evaluation was performed using the genism and spaCy libraries in Python (Honnibal & Montani, 2017; Rehurek & Sojka, 2011).

Word embedding approaches, such as the popular word2vec algorithm, transform concepts into vectors in high-dimensional space based on their co-occurrence within a corpus (Mikolov et al., 2013). The semantic similarity between two concept vectors can then be captured by computing their cosine similarity, or the inverse of the cosine distance between the two vectors. The accuracy of these vectors depends on the size of the corpus they are trained on, but fortunately a number of high-performing pretrained vectors are now available (Rodriguez & Spirling, 2022). For this study, we utilized the ConceptNet embeddings, which have been particularly tuned for accurately representing semantic relations (Speer et al., 2017).

Word embeddings enable the automated retrieval of relevant terms (theology, creator, creation) using a single seed term that does not need to be pre-specified before conducting the analysis. This has an advantage over traditional word count approaches is that all words with a semantic relation to the token are exhaustively assessed, regardless of differential spellings or conjugation. Depending on the size and accuracy of the pretrained vectors, embeddings enable retrieval of more obscure terms of interest, which can correct for the bias of a typical dictionary creator towards either very common words or words that they often encounter due to their social positionality or occupation. Of course, word embeddings carry their own sets of biases based on the texts they are trained on. ConceptNet embeddings perform better in general on these issues due to their incorporation of high-level semantic knowledge and ontology, rather than only creating embeddings based on large corpora alone. This allows for more “official” relations of meaning between words to be represented with the trade-off of some of the texture of everyday popular use and colloquialisms, but we found these embeddings to be the most accurate and consistent in dealing with the rare and abstract words that often accompany supernatural experiences.

Once our dictionaries were constructed, we used them to conduct word counts, similar to the process found in LIWC studies. Each narrative was tokenized and stop words were removed. Narratives with less than 5 tokens remaining were then removed. Words were scored by semantic similarity to averaged vectors from each dictionary using the standard metric of cosine similarity. A word was counted for a dictionary if it had the highest score (thus avoiding overlap between dictionaries) and if it was above a threshold of cosine similarity 0.5. After extracting these tokens, we then constructed contextualized embeddings in order to only select narratives with the intended use-case of the token in question (i.e. omitting references to God in interjections like “Oh my God!”).

In the second part of the analysis, passages were created from counted tokens using the sentence they were contained in, plus the surrounding sentences. These were scored using vectors created through the method described above. Dimensional projection is useful because strong antonyms (good/bad, up/down) are often quite close together in a word embedding space due to semantic similarity. By comparing our passages or words of interest to the averaged difference of the antonym pairs, we get scores that are either positive or negative depending on their proximity to either “pole” of the dimension. Scored passages were then evaluated using ANCOVA to control for passage length between our groups of interest. After evaluation, these features were then used to construct our supernatural text classifier for our primary study.

When searching for the occurrence of “supernatural” relevant semantic domains, vectors retrieve a more complex result than word counts. For example, if we take the following sentence below and score the cosine similarity to the word “God”, we get the following:

“In (.05) the (.1) light (.08) of (.15) the (.1) "New (.04) Theology (.38)" and (.04) my (.19) studies (.05) as (-.01) a (.04) scientist (.04) I (.14) realise (.06) that (.12) one's (.06) early (-.02) conceptions (.18) of (.15) God (1) the (.1) creator (.41) must (.0) be (.08) modified (0) as (-.01) we (.14) learn (-.04) more (0) of (.15) the (0.1) continuing (-0.07) process(.07) of (.15) creation (.33).”

Here we see that cosine similarity does not just capture the target word in question, but also semantically similar words (Theology, creator) that are not specified ahead of time. This effect can be enhanced by creating mean vectors from many words in the target area, which creates a vector that roughly captures the semantic space between those words. Text can then be searched by multiple dictionaries by first only selecting the tokens above a certain semantic threshold and then by assigning them to the dictionary with the highest cosine similarity score (forced choice).

In order to get the most efficient semantic retrieval, we started with seed words related to supernatural agents and religious agents. We then requested from ConceptNet clusters of words closest in the semantic space for each term, and then clustered and mapped all the terms to let inductive semantic clusters emerge. These were then narrowed based on relevance. We also constructed several dictionaries with the intent of filtering out irrelevant words, such as a “proper nouns” dictionary so as to not retrieve common names with a religious origin (Joshua, Michael, etc.). This yielded the following dictionaries (supplementary table 2)

After tokens were retrieved with the context of surrounding sentences, we then generated contextual embeddings for the tokens using a transformers-based library (Song et al., 2020) which creates representations that can capture subtle differences between word usage (for example between “oh hell!” as an exclamation and “I went to hell” as a place). These embeddings were then reduced using Uniform Manifold Approximation and Projection and clustered using Hierarchical Density-Based Spatial Clustering of Applications with Noise (HDBSCAN) in a method similar to that employed by the BERTopic library (Grootendorst, 2022). A visualization of one such cluster for words related to “magic” in dream narratives is depicted in supplementary figure 1.

Supplementary table 3 demonstrates the semantic variability between two clusters even when the same word is employed. In this context, we would accept cluster 7 as relevant and delete cluster 8 from the search. We iterated through each dictionary of interest until all irrelevant clusters were excluded. This yielded the following counts of tokens (3,466 counts of tokens involving supernatural content across 2,457 narratives; supplementary table 4).

We then scored the narratives by creating averaged vectors from one side of a semantic dimension (such as “Fast”) and subtracting the averaged vector from the other side (such as Slow), which then can assess averaged vectors from text along a single intended dimension. Dictionaries used to construct dimensional vectors are on supplementary table 5.

We next used our cognitive dictionaries to compare dream narratives with supernatural content to dreams without supernatural content. We found that supernatural tokens in dream content were associated with more semantic dimensions of unfamiliarity and negative emotionality compared to dream content without supernatural tokens. These features provided the means for training the LightGBM text classifier (see main text section 2.2.1). We also trained a single-label classification model using the simple-transformers library (Wolf, 2020).

***Study 1 Methods***

We had an additional 35 participants start the study but not complete it: 26 participants withdrew for a variety of personal reasons and 9 participants were removed from the study for missing too many study activities (e.g. not consistently filling out morning questionnaires or diaries etc.). Participants received the first link manually, then the system automatically sent subsequent links upon survey completion. Researchers monitored survey submissions and reached out with survey links and reminders if participants did not submit at their usual times (participants were instructed to follow their normal sleeping schedule, so these times varied).

We sent out an additional 25 Dreem 3 Headbands (DHs), but participants discontinued usage for a variety of reasons. Participants were sent the DH along with Velcro extensions, an elastic sweatband, and a charger via USPS to their home, then met with a researcher over Zoom for a tutorial on how to properly wear it to achieve the best signal quality, including using the live signal check feature of the app.

From the resulting 896 recordings from 67 participants, we excluded data from nights where “proportion on head and scorable” was below 65% or channel quality for F7-F8 was below 50% (149 recordings). Then, all data from participants with less than 7 nights of good quality remaining was removed (6 participants, 28 recordings). Additionally, 12 DH recordings were excluded either because participants alerted their research contact that they started the recording part way through the night, or they did not have corresponding morning surveys. This left us with at least 7 nights of quality recordings from 61 participants for a total of 707 usable recordings. These remaining DH recordings had an average of 89.18% (SD=9.18) “proportion on head and scorable” and an average F7-F8 channel quality of 87.76% (SD=9.87).

***Study 1 Materials - Sleep Architecture***

The DH has been previously validated against gold standard polysomnography (PSG) and sleep staging scoring experts in multiple peer reviewed publications utilizing healthy volunteer adults including: Chinoy et al. (2022), N = 21; Thorey et al. (2019), N = 25; Thorey et al. (2020), N = 42; Birch (2021), N = 10; and Arnal et al. (2020), N = 25, where DH detection and automatic sleep staging was compared against PSG and sleep EEG manual scoring staging experts. In 5 of these 6 studies, DH staging achieved an accuracy of at least 80% for all sleep stages (N1, N2, N3 and REM) studied; one reached only 75% (Birch, 2021).

The DH has successfully been utilized in well over 30 studies across very diverse populations including people during COVID lockdown (Pépin et al., 2022), Parkinson’s (Anjum et al., 2024; González et al., 2024), Alzheimer’s (Muurling et al., 2021), PTSD (Saguin et al., 2021), nightmares (Schwartz et al., 2022), sleep apnea (Waeber et al., 2021), narcolepsy (Asp et al., 2022), and of course healthy volunteers.

The DH is made of foam and fabric with an elastic band with optional Velcro extensions to ensure a secure fit for different head sizes. The DH contains embedded sensors including frontal (F7 and F8) and occipital (O1 and O2) yielding derivations F7-O1, F8-O2, F8-F7, and F8-O1 (bandpass Butterworth order 2 between 0.4Hz and 25Hz. 3 additional notch filters are added to remove 50Hz and 60Hz electrical noises, and 62.5Hz). To find cycles per minute, an accelerometer tracks movements on the x, y, and z axes for 3 channels to calculate breathing rate, which go through a band pass filter (0.1Hz, 0.5Hz), followed by PCA to identify the component of greatest variability, and zero crossing detection. A bone conduction speaker delivers audio messages to the wearer when recordings are started and stopped. The DH is used in conjunction with the ‘Alfin’ application, freely available on Android and Apple iOS, which allows the transfer of stored data via Bluetooth. Participants were provided with app log-in details to ensure full data confidentiality and anonymity. Users can view metrics regarding their night’s sleep on the app and researchers can access the DH servers to see data that the algorithm has automatically scored.

***Study 1 Measures - Dream collection***

The first question on the morning survey asked participants to report any dream content they could recall from the entire night. A later prompt asked participants to focus on the most impressive dream from the night and write it out in detail; they are asked one final time for any additional content they recalled while completing more ratings. In order to analyze dream content, these reports were cleaned for typos, abbreviations were expanded, and reports were separated into individual dreams. For dreams that had content reported multiple times, the reports were combined to include all relevant information. Any text not directly related to the dream experience was removed (for example “I had a dream that…” or “I think I remember that…” or “but in real life it’s actually…”) in order to avoid these words being included in automated text analysis as part of the dream experience.

***Study 1 Data Analysis***

We first tested the dimensions of baseline religiosity for impacts on morning closeness to god during the longitudinal study (supplementary table 6). Of the five factors considered, only BMMRS Positive Spirituality was significant, indicating that that factor is also a valid measure of personalist theism in general. Based on this model, we only retained BMMRS Positive Spirituality from the BMMRS for our further modeling.

We then also tested the Revised Paranormal Belief Scale sub-factors for effects on morning closeness to God. While some studies have found a relationship between the RPBS and theistic beliefs, the correspondence between the two is far from well-established (Pennycook et al., 2012). Our mixed effects models found a significant relationship between Traditional Religious Belief and Spiritualism and morning closeness to God, but no other factors (supplementary table 7). The role of spiritualism here follows logically from longstanding theoretical work in the cognitive science of religion that designates mind-body dualism as a key component of theistic beliefs (see the Discussion in the manuscript).

Further testing of these three factors together revealed that BMMRS Positive Spirituality has a suppression effect on TRB and Spiritualism (supplementary table 8). Based on visual inspection of BMMRS Positive Spirituality (supplementary figure 2), we then considered a random-slopes model as well as random-intercepts. A random slopes model for both Spiritualism and BMMRS Positive Spirituality failed to converge, so our final model only included random slopes for BMMRS Positive Spirituality (table 2 in the manuscript).

***Study 2 Methods***

*ANN basics: The Perceptron and Multi-Hidden Layer Perceptron*

The fundamental unit of the artificial neural network (ANN) is the perceptron. A perceptron is a node in an ANN that takes inputs, applies a mathematical combination of the inputs with a bias/intercept (e.g. linear combination), and puts the output through a (usually nonlinear) activation function. An often-used activation function is ReLU, which transforms the perceptron output to 0 if the perceptron output is negative, or in other words applies the transformation max(0, perceptron_output).

Perceptrons are often organized in layered networks, with an input layer, an output layer, and one or more hidden layers. When set up as feedforward networks, information flows through the network of perceptions in a single direction from inputs to outputs (no looping). The closeness of ANN outputs to a pre-specified target is used to evaluate biases and weighting factors. Computational power permitting, a comprehensive survey of biases and weighting factors across the entire space of inputs and outputs yields the optimal ANN – a model that replicates input-output relationships in the dataset as closely as possible. Feedforward ANNs have been heavily researched (Hornik et al., 1989) due at least in part to the key mathematical result demonstrating that “standard multilayer feedforward networks are capable of approximating any measurable function to any desired degree of accuracy … any lack of success in applications must arise from inadequate learning, insufficient numbers of hidden units, or the lack of a deterministic relationship between input and target” (Hornik et al., 1989). This indicates the modeling potential of ANNs, which far exceeds that of linear regressions, so long as there is sufficient training data and an appropriate ANN design.

Back-propagation further increases the power of ANNs by using the precise magnitude and distribution of errors in the output layer (compared to the pre-specified target data) to rejigger weights and biases first in the ANN’s output layer and backwards through all prior layers to the first hidden layer that processes input data. Caruana (1997) first established that back-propagation ANNs can simultaneously learn multiple tasks in parallel while also learning trends that are generalizable across the tasks. Zhang & Yang (2021) survey several real-world applications of multi-task learning to boost ANN performance, which increases computational efficiency and expands the modeling reach of ANNs even further beyond what linear regression can accomplish (Caruana, 1997).

# Supplementary Figures and Tables

**Supplementary Figure 1.**

***
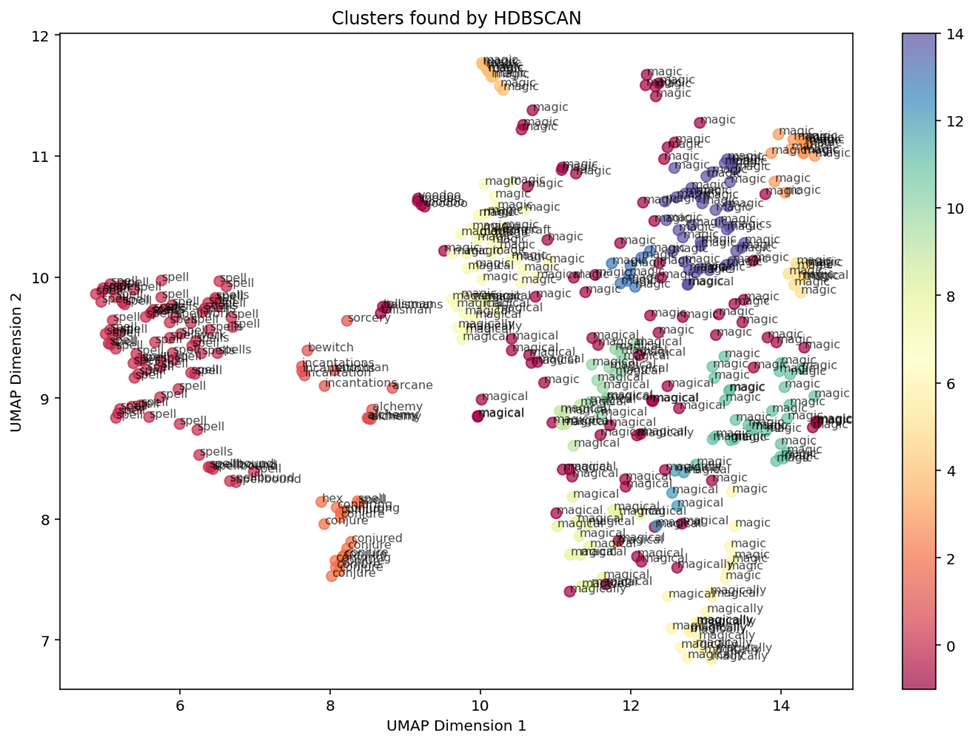
***

*Supplementary Figure 1: Two-dimensional representation of contextual embeddings of magic-related terms in dreaming*. This figure demonstrates the two-dimensional projection using Uniform Manifold Approximation and Projection, a high-performing algorithm that can map high-dimensional data in lower-dimensional space while preserving many features of data structure. Cluster colors are assigned by Hierarchical Density-Based Spatial Clustering with Noise (HDBSCAN), an algorithm that can assign observations to clusters without pre-specifying a number of clusters *(46)*. Proximity in between words corresponds to semantic similarity between contextual embeddings

**Supplementary Figure 2.**

***
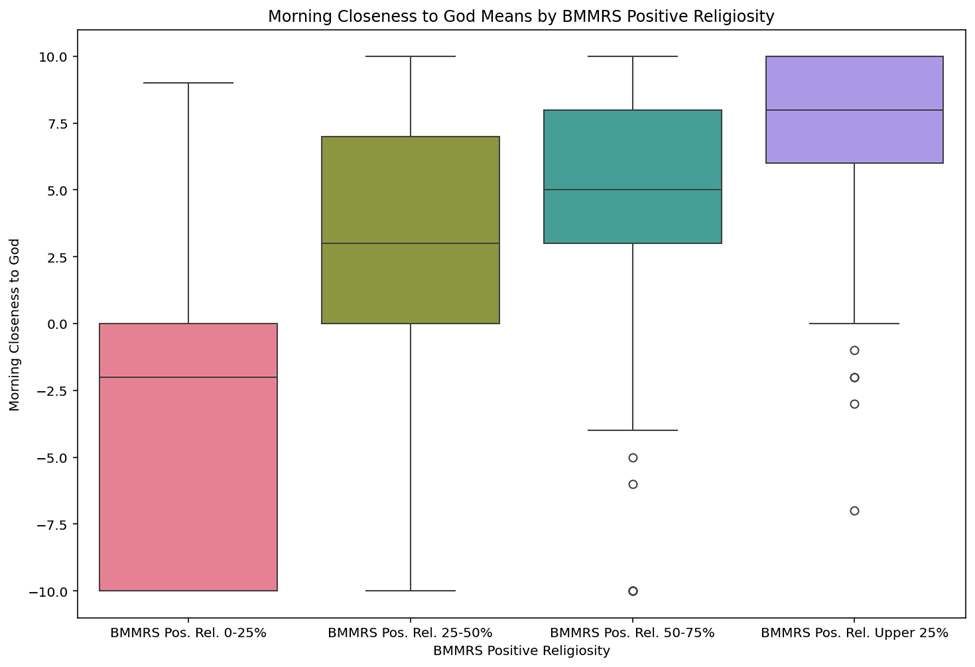
****Supplementary Figure 2: Boxplots of Closeness-to-God Means Grouped by BMMRS Positive Spirituality.* This figure visually represents the relationship between baseline scores of the BMMRS subfactor of Positive Spirituality and participant means on the longitudinal morning measure on closeness to God. Bins of four were chosen for demonstrative purposes and do not correspond to statistically defined groupings.

**Supplementary Figure 3.**

**
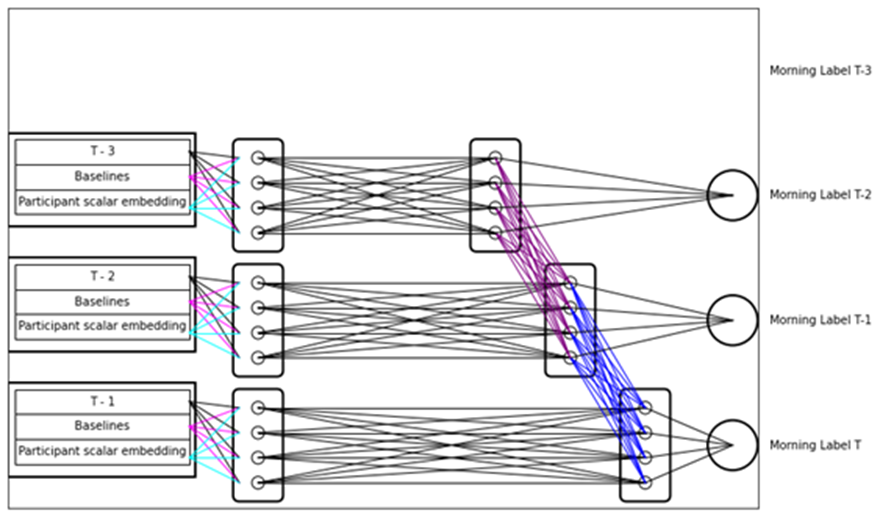
**Supplementary Figure 3: Three-day lag network

**Supplementary Figure 4.*
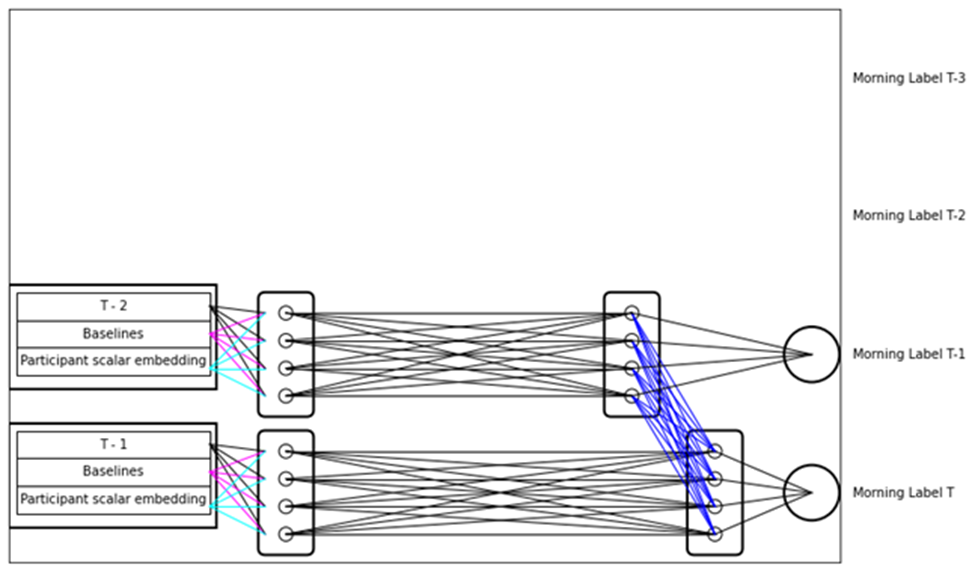
***Supplementary Figure 4: Two-day lag network

**Supplementary Figure 5.*
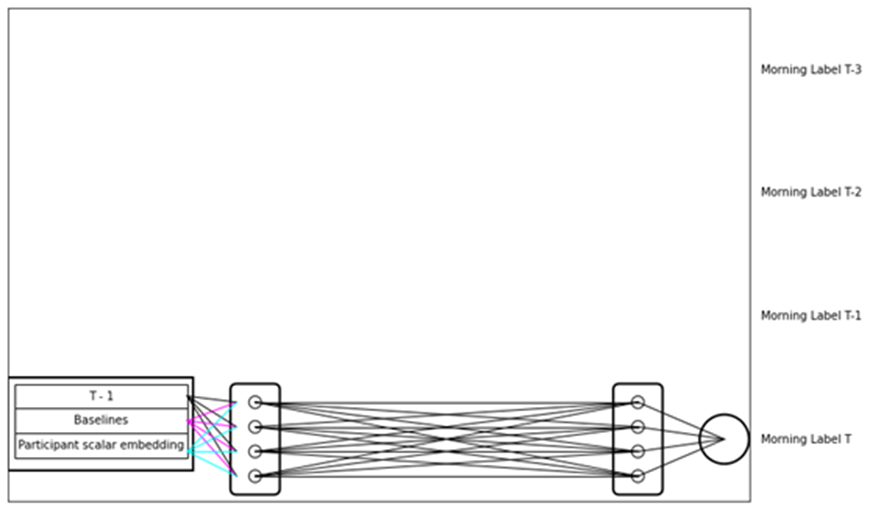
***Supplementary Figure 5: One day lag network

**Supplementary Figure 6: Marginal Effects Plot of Interaction between Spiritualism and Dream Affect on Morning Closeness to God
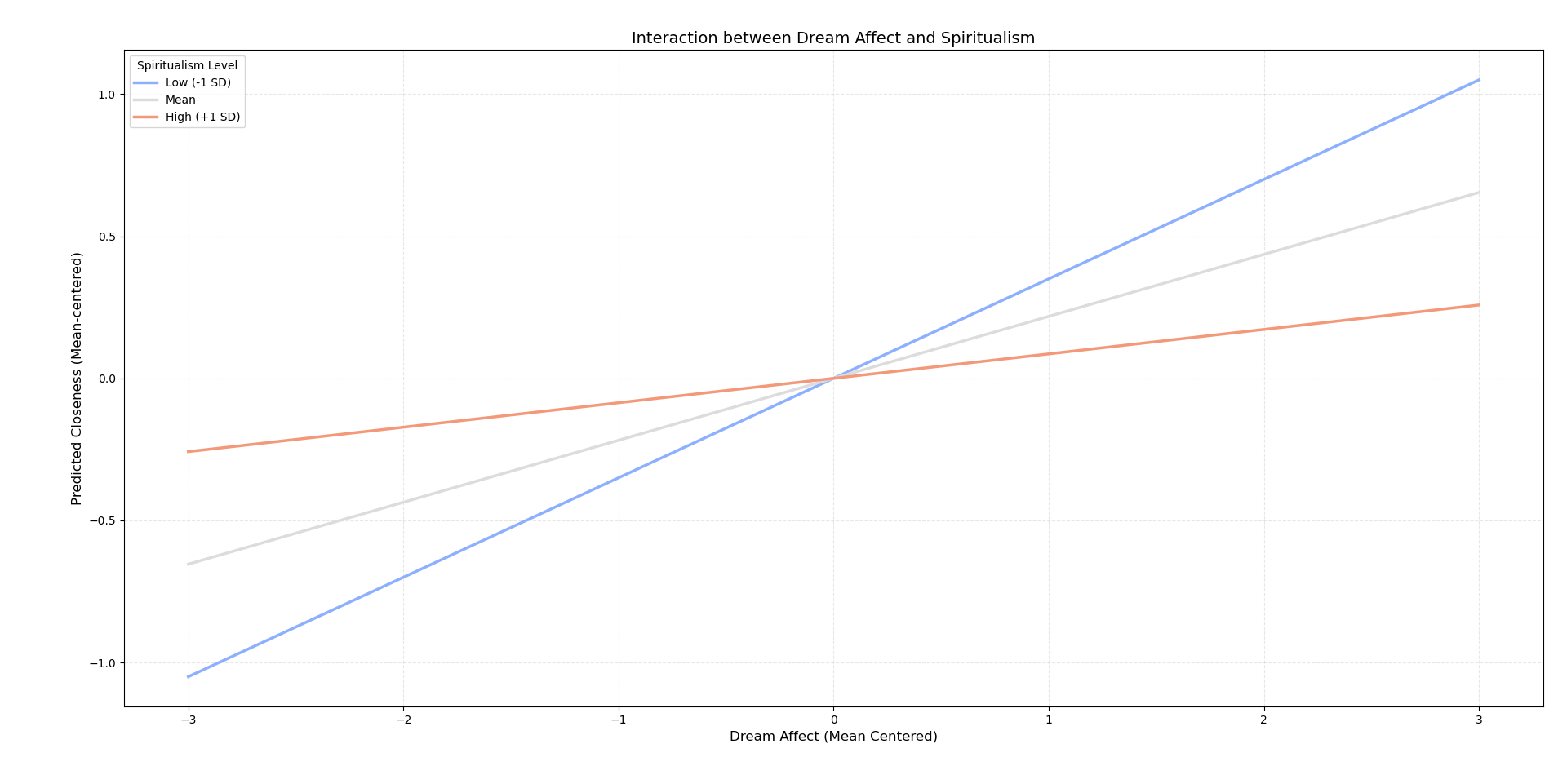
**
Supplementary Figure 6: A Marginal Effects Plot of the Relationship between Spiritualism and Dream Affect on Closeness to God based on a Linear Mixed Effects Model

**Supplementary Table 1: Dimensional Scores of Supernatural Dream Narratives**

|  | **Sup. dreams** | **Control: Non-sup. dreams** | | |
| --- | --- | --- | --- | --- |
| **Semantic Dimension** | **Mean** | **Mean** | **F** | **p** |
| Valence (Positive-Negative) | -0.047 | 0.003 | 5.376 | 0.02 |
| Arousal (Positive-Negative) | -0.035 | 0.024 | 5.166 | 0.023 |
| Dominance (Dominant-Submissive) | -0.059 | -0.264 | 64.135 | <.001 |
| Gender (Male-Female) | -0.035 | 0.029 | 4.735 | 0.03 |
| Size (Big-Small) | 0.014 | -0.154 | 44.898 | <.001 |
| Affiliation (Friendly-Hostile) | -0.172 | 0.021 | 68.993 | <.001 |
| Familiarity (Familiar-Strange) | -0.23 | 0.081 | 176.486 | <.001 |
| Openness (Open-Hidden) | -0.333 | -0.016 | 164.773 | <.001 |
| Loudness (Loud-Quiet) | 0.216 | 0.444 | 85.457 | <.001 |
| Desire (Desiring-Rejection) | -0.122 | -0.179 | 6.297 | 0.012 |
| Power (Powerful-Powerless) | 0.074 | -0.159 | 89.507 | <.001 |
| Effort (Effortless-Effortful) | -0.256 | -0.454 | 69.411 | <.001 |
| Proximity (Near-Far) | 0.106 | 0.279 | 44.734 | <.001 |
| Speed (Fast-Slow) | 0.054 | 0.03 | 1.81 | 0.179 |
| Control (Controlled-Chaotic) | -0.005 | 0.172 | 71.914 | <.001 |
| Protection (Protect-Attack) | 0.111 | 0.037 | 6.575 | 0.01 |
| Planning (Planned-Improvised) | -0.003 | 0.121 | 30.83 | <.001 |
| Colorfulness (Colorful-Colorless) | 0.16 | -0.094 | 103.345 | <.001 |
| Position (Above-Below) | 0.031 | 0.062 | 1.083 | 0.298 |
| Direction (Forward-Backward) | -0.243 | 0.018 | 106.713 | <.001 |
| Movement (Movement-Static) | 0.245 | 0.123 | 18.583 | <.001 |
| Location (Inside-Outside) | -0.301 | -0.234 | 7.113 | 0.008 |
| Temporal (Future-Past) | 0.123 | 0.051 | 14.898 | <.001 |
| Substance (Solid-Insubstantial) | 0.235 | 0.536 | 212.471 | <.001 |
| Virtue (Virtue-Vice) | -0.559 | -0.331 | 125.608 | <.001 |
| Life-Death (Life-Death) | -0.173 | -0.3 | 39.08 | <.001 |

**Supplementary Table 1. ANCOVA comparisons of supernatural dreams**. Supernatural dreams (Sup. dreams) compared to control narratives: non-supernatural dreams (Non-sup. dreams). Means are calculated from values that have been scaled using Z-score normalization for the entire dataset. Significance is assessed at ɑ < .001 with the Bonferroni correction for multiple comparisons.

**Supplementary Table 2. Semantic Dictionaries for Retrieval of Supernatural Content**

| afterlife | after_life, afterlife, beforelife, life_after_death, afterlives, next_world, afterworld, next_life, kingdom_come, great_beyond |
| --- | --- |
| angel | angelly, angelization, angelize, angellike, nonangelic, angelify, angelist, angel, unangelic, angelism |
| animals | animal, horse, bear, deer, dog, cat |
| astral body | astral_body, astral, astral_projection, devachanic, astrally, astral_plane, devachan, subastral, spiritual_world, phantasmology |
| beast | beast, unbeast, beastling, beasten, beasthood, beastdom, theriologic, bebeast, beasts, beastlike |
| bogeyman | bogeyman, bogyman, boogyman, boogeyman, boogieman, bogeywoman, bugbear, bugaboo, whangdoodle, bug_boo |
| heaven | celestial_city, heavenize, heaven, city_of_god, heavenlike, heavenric, heavenish, pearly_gates, holy_city, cyberheaven |
| centaur | centaur, centauress, hippocentaur, centaurette, centauroid, onocentaur, centauresque, centaurian, centaurs, epona |
| chimera | chimera, chimeralike, chimeral, chimaeriformes, chimaera, chimerical, mythical_monster |
| creature | creature, creatures, birds_and_animals, creatureless, creaturize, werecritter, creatural, merbeast, critter, theriomorphic |
| cryptid | cryptid, skunk_ape, sasquatch, hairy_thing, unshaved_thing, loch_ness_monster, abominable_snowman, chupacabras, chupacabra, tatzelwurm |
| curse words | damn, shit, goddamnit, heck |
| devil | devil, devilet, demidevil, devilship, devility, devilkind, devilism, deviling, devildom, old_gooseberry |
| devilish | devilish, diabolicalness, diabolical, devilishness, mephistophelean, diabolic, diabolicality, mephistophelian, fiendish, diabolize |
| djinn | djinn, djinni, jinn, jinnia, muslim_demonology, jinni, genies, ifrit, efreet, afrit |
| earthish | earthish, sublunary, earthhood, superterrestrial, terrenely, earthly, sublunar, earthliness, subterrestrial, sublunary_sphere |
| elf | elf, elves, elfkin, elfen, elven, wood_elf, light_elf, dark_elf, elvish, elfe |
| evil | evil, sexual_immorality, eviler, malefactory, ponerology, evilest, felonous, evils, maleficent, think_cats |
| exorcizer | exorcizer, exorciser, exorcizing, drive_out_devils, exorcising, exorcistical, exorcise, exorcize, exorcism, exorcistic |
| extradimensional | extradimensional, interdimensional, transdimensional, otherdimensional, hyperspatial, wormholes, faster_than_light, holoprojection |
| faery | faery, fairy, fairydom, cailleach_bheur, faerie, féerie, fairily, fairies, faeries, piskie |
| fairy tale | fairy_tale, fairytale, fairy_story, fairy_talelike, nursery_tale, fairytales, fairy_tales, fairybook, fablelike, big_bad_wolf |
| fiend | fiend, fiendom, fienddom, fiendful, fiendkin, fiendling, fiendlike, fiends, fiendly, sex_fiend |
| freak | freak |
| ghost | ghost, ghostling, ghostkind, ghostology, ghostologist, ghostological, ghostsome, vengeful_spirit, ghosten, ghostess |
| ghostish | ghostish, ghostly, unghostly, ghostlily, nonghostly, spectred, spectrality, specterlike, ghostlike, unspectral |
| ghoul | ghoul, ghouls, ghoulification, ghoulishness, ghoulie, ghast |
| goblin | goblin, goblinry, gobliness, hoblin, goblinize, skratte, hobgoblin, iberian_folklore, goblindom, bugan |
| goddess | goddess, goddessship, goddesship, goddess_ship, goddesshood, deess, female_deity, goddesslike, goddessling, triple_goddess |
| guru | guru |
| hell | hell, hellward, hells, hellsome, hell_on_earth, ceaseless_pain, malebolge, helldoomed, hellbred, helly |
| hindu mythology | hindu_mythology, brahmarakshasa, asura, rakshasa, yaksha, meghanada, daeva, utukku |
| japanese mythology | japanese_mythology, enma, kagutsuchi, shinigami, tutelary_deity, tengu, yokai, magical_girl, otakukin, watches_over |
| dragon | linnorm, dragon, lindorm, pseudodragon, lindworm, dragonise, chinese_dragon, dragonlike, dragoness, lindwyrm |
| zombie | living_dead, zombie, zombyish, zombiism, undead, horror_fiction, zombi, draugr |
| mermaid | mergirl, merperson, merwoman, mermaid, mermaidlike, mermaids, merfolk, merlady, merkid, ethel_merman |
| monastery | monastery, religious_residence, monasterial, christian_monastery, friary, convent, archabbot, antimonastic, monachization, archabbey |
| monster | monster, monsterling, monsterlet, monsterlike, monsterise, xiangyao, monstrification, monstery, kanghui, supermonster |
| monstrous | monstrous, monstruous, monstrocious, monstrousness, monstrosity, immane, monstrously, monstrosities |
| mythic figures | asgard, jupiter, apollo, thor, zeus, hercules, hades, gaia, osiris, eleusinian, jove, heimdallr, perseus, ares, dionysus, odysseus, themis, sekhmet, theseus, athena, orpheus, aphrodite, kore, kratos, horus, agamemnon, cronus, demeter, hyperion, danae, anubis, minerva, odin, quetzalcoatl, orphic, eirene, loki, balder, poseidon, valhalla, graeco_roman_deity, greek_god, mythological_figure, roman_god, greek_deity, norse_deity, sun_god, orisha, kami |
| necromancy | necromance, necromancer, necromancy, necyomancy, psychagogue, chinese_shawm, necromantic, sciomancer, psychomanteum, psychomancy |
| netherworld | netherworld, nether_world, nether_worlds, netherworlds, realm_of_dead, underworld, underworldly, world_of_dead, nether_regions, nether_region |
| nymph | nymph, water_nymph, salmacis, hydriad, woodnymph, nymphoid, dragonfly_naiad, water_sprite, orphne, dryad |
| ogre | ogre, ogreish, ogres, ogry, trolless, trollette, orcess, goblette, ogrish, ogress |
| supernatural | paragnosis, supernatural, supernaturalize, supernaturalization, supernaturalise, supernaturality, transmundane, omnimalevolent |
| paranormal | paranormal, paranormalist, paranormality, parapsychological, psychometer, golden_dawner, paranormally, psychic_phenomena, x_filesy, parapsychology |
| phantasmal | phantasmal, phantasmatical, phantasmic, phantasmally, phantasmically, phantasm, phantasms |
| phrygian_deity | phrygian_deity, persian_deity, egyptian_deity, chinese_deity, roman_deity, semitic_deity |
| piskie | piskie, sentient_animal, ouphe, mythical_being, fictional_thing, supernatural_being, good_folk, imaginary_being, gog_and_magog, faeling |
| poltergeistic | poltergeistic, poltergeist, poltergeisty, poltergeistlike, poltergeists, poltergeistism |
| proper_noun_catch | josh, kyle, matt, james, carol, mary |
| psychic | psychic, spirit_rapper, nonpsychic, psychicist, psychicism, parapsychic, psychics, telepsychic, geokinesis, biopsychic |
| satan | satan, satanian, prince_of_darkness, evil_one, satanize, satanical, theistic_satanism, father_of_lies, fallen_angel, lord_of_flies |
| sea_monster | sea_monster, merhorse, ophiotaurus, sea_serpent, kraken, lusca, kraken_mare, pixiu, hircocervus |
| sky | sky, clouds, sun |
| soothsayer | soothsayer, fortune_teller, foreteller, fortuneteller, völva, seeress, soothsaying, fortune_tellers, seer, farseer |
| sorcerer | sorcerer, magic_user, vitki, archimage, spellcaster, ensorcel, mage, wizard, wizardess, warlock |
| spiritism | spiritism, spiritualism, spiritualist, spiritualistic, kardecism, spiritist, psychographer, ghostdom, psychographist, spiritistic |
| spook | spookery, spooker, spook, spooky, outspook, spookfest, spookable, spooktastic, spooktacular, creeptacular |
| stygian | stygian, acherontic, subtartarean, acheronian, tartarean, stygobiont, avernus |
| supreme_being | supreme_being, deity, male_deity |
| teleportation | teleportage, teleport, unteleported, teleported, teleporter, teleports, teleportal, teleportation, teleporting, beam_up |
| thermokinesis | thermokinesis, telekinetics, macrokinesis, telekinetic, telekinesis, microkinesis, psychokinesis, psychokinetically, macrokinetic, pyrokinetic |
| transmedium | transmedium, mediumship, mediumist, mediumistic, female_fortuneteller, child_from_city, mediumic, spirit_medium, channeler, mediums |
| ufo | ufo, unidentified_flying_object, ufos, ufology, unidentified_flying_objects, tall_white, ufologist, ufologists, flying_saucer, close_encounter |
| unearthliness | unearthliness, otherworldly, unearthly, otherworldy, other_worldly, eldritch, ultramundane, eldritchly, uncanny, otherworldliness |
| vampire | vampire, vampirelike, vampiristic, nonvampire, vampiresque, oupire, vampirehood, vampiredom, vampirisation, vampirina |
| werewolf | werewolf, lycanthrope, werewolfdom, werewolfish, loup_garou, were_wolf, werewolfism, werewolves, turnskin, lycanthropic |
| witch | witch, soucouyant, nonwitch, unwitch, witchly, craig_flounder, witchy, black_witch, witchlet, witches |
| wizardcraft | wizardcraft, goety, black_art, sorcery, warlockry, sorceries, pishogue, sorcerously, witchcraft, goetic |
| wolf | wolf, lupicide, wolvish, wolf_eliminator, tibetan_wolf, hugo_wolf, friedrich_august_wolf, white_wolf, wolfly, gray_wolf |
| wraith | wraith, wereghost, draug, boggart, revenant, fantom, barghest |
| zombie | zombie |

Supplementary Table 2: These dictionaries refer to the terms that were used to create averaged vectors for the extraction of supernatural tokens from dream and religious/spiritual experience narratives.

**Supplementary Table 3. Example Comparison Between Contextual Embedding**

| Token | Context | HDBSCAN Cluster |
| --- | --- | --- |
| magical | I dreamt I was being chased by some bad magical creatures. I had a vehicle that was defeating them and carrying me to safety. Like I was Harry Potter. | 7 |
| magical | I steer the boat towards the wave, curving up inside it as the top begins to curl down….wow, it is an amazing, magical sight, inside the vast space of the hole within the wave | 8 |

Supplementary Table 3: This table gives example comparisons between two clusters defined by contextual embeddings of the same token (“magical”). Sentence contexts indicate the different uses of the term (“magical creatures” referring to entities with supernatural abilities and “an amazing, magical sight,” synonymous with wonder and beauty). This clarifies how our term discarded uses of supernatural-like tokens referring to other contexts.

***Supplementary Table 4. Count of Supernatural Tokens***

| **Count** | **Token(s)** |
| --- | --- |
| 235 | space |
| 162 | magic |
| 145 | monster |
| 142 | ghost |
| 140 | alien |
| 126 | aliens |
| 123 | creature |
| 111 | angel |
| 102 | witch |
| 91 | heaven |
| 77 | dragon |
| 69 | haunted |
| 67 each | magical, devil |
| 66 | ghosts |
| 64 | zombies |
| 61 | vampire |
| 59 | monsters |
| 51 | spell |
| 49 | zombie |
| 46 | mermaid |
| 45 | psychic |
| 44 | spaceship |
| 41 | demon |
| 37 | fairy |
| 32 | vampires |
| 30 | god |
| 28 | goddess |
| 26 | witches |
| 25 | wizard |
| 23 | hell |
| 21 | magically |
| 18 | dragons |
| 17 each | angels, beings |
| 16 each | universe, esp |
| 15 | telepathically |
| 13 each | mermaids, critters |
| 12 each | prophetic, gods, elf, reptile |
| 10 each | precognitive, spaceships, witchcraft, spells, fairies |
| 9 each | realm, heavens, conjure, creatures |
| 8 each | premonition, divine, oracle, astral, mystic, tarot, heavenly, paradise, levitate, disembodied, levitating |
| 7 each | telepathic, pegasus, elves, critter, pixie |
| 6 each | superpowers, teleport, teleported, parapsychological, werewolf, astrologer |
| 5 each | telekinesis, angelic, ghostly, potions, psychically, centaur, potion, deities |
| 4 each | voodoo, unicorns, leprechaun, gnome, paranormal, spellbound, leprechauns, alchemy, telepathy, prophecy, parapsychology |
| 3 each | predict, enchanted, predictions, metaphysical, esoteric, genie, prescience, spacecraft, witchy, goblins, phenomena, dimensions, underworld, teleportation, fae |
| 2 each | enchanting, psychokinesis, werewolves, forecast, hag, incantations, prophesy, predicts, conjuring, broomsticks, godly, deity, spellwork, mage, poltergeist, seer, ghostbusters, corporeal, celestial, reptilian, hobbit, princesses, haunting, minotaur, summoning, realms, trolls, supernaturally, superhuman, parapsychologist, zombified, clairvoyant, seers |
| 1 each | Tinkerbell, psychics, universes, galactic, starship, multidimensional, interstellar, fairyland, prophesied, hex, bewitched, hags, faeries, faerie, ghostbusting, hauntings, premonitions, conjured, shapeshift, orcs, zombocalypse, zombielike, incorporeal, magics, talismans, enochian, sorcery, clairvoyance, arcane, divining, incantation, mothman, bigfoot, talisman, devils, apparitions, shapeshifter, warlock, nether, teleports, satyr, troll, ogre, clairvoyantly, ogres, omens, psychical, occult, wizards, prophesying, runes, enchants, supernormal, pooka, teleporter, teleporting, precognition, netherworld, apparition, goblin, gremlin, goddesses, purgatory, dragonet, fortuneteller, telekinetic, precog, prediction, psychokinetic, titans, titan, necromancer, foretold, basilisk |

Supplementary Table 4: This table reports the counts of each supernatural token type for our analysis overall across all dream narratives.

**Supplementary Table 5. Antonym Terms for Dimensional Vectors**

| Dimension | Positive Pole | Negative Pole |
| --- | --- | --- |
| Valence | good, happy, satisfied, hopeful | bad, unhappy, unsatisfied, disgruntled |
| Arousal | engaged, entertained, excited | bored, unamused, calm, sluggish |
| Dominance | dominant, powerful | weak, submissive |
| Gender | male, man, masculine | female, woman, feminine |
| Size | big, giant, huge | small, tiny, little |
| Affiliation | friendly, safe, benevolent | hostile, dangerous, menacing |
| Familiarity | familiar, regular, everyday | strange, odd, unfamiliar |
| Openness | open, unconcealed, exposed | hidden, concealed, veiled |
| Loudness | loud, shout, roar | quiet, silent, hushed |
| Desire | desiring, wanting, craving | rejecting, dismissive, deny |
| Power | powerful, mighty, potent | powerless, weak, impotent |
| Effort | effortless, fluent, graceful | effortful, clunky, hesitant |
| Proximity | near, close, adjacent | far, distant, remote |
| Speed | fast, rapid, quick | slow, slowness, sluggish |
| Control | controlled, regulated, organized | chaotic, uncontrolled, wild |
| Protection | protect, defend, safeguard | attack, hurt, harm |
| Planning | planned, intended, premeditated | improvised, spontaneous, unplanned |
| Colorfulness | colorful, multihued, prismatic | dull, drab, colorless |
| Position | above, top, higher | below, beneath, lower |
| Direction | forward, ahead, front | backward, behind, reverse |
| Movement | movement, motion, dynamic | static, sit, still |
| Location | inside, within, interior | outside, outer, exterior |
| Temporal | future, tomorrow, later | past, yesterday, earlier |
| Substance | solid, dense, thick | insubstantial, transparent, ephemeral |
| Virtue | virtuous, patient, compassionate | vice, greedy, cruel |
| Life-Death | life, alive, living | death, dead, deceased |

Supplementary Table 5: This table shows the antonym terms used to construct dimensional semantic vectors in order to score the averaged semantic vector of each passage along a single dimension.

**Supplementary Table 6: Mixed Effects Linear Regression Model Predicting Closeness-to-God from BMMRS Factors**

|  | Estimate | 2.5_ci | 97.5_ci | SE | T-stat | P-val | Sig |
| --- | --- | --- | --- | --- | --- | --- | --- |
| (Intercept) | -5.057 | -7.424 | -2.838 | 1.166 | -4.337 | <.0001 | *** |
| BMMRS Positive Spirituality | 6.434 | 4.796 | 7.994 | 0.835 | 7.707 | <0001 | *** |
| BMMRS Religious Practices | -0.305 | -1.746 | 1.074 | 0.73 | -0.418 | 0.676 |  |
| BMMRS Positive Congregational Support | -0.267 | -1.229 | 0.748 | 0.509 | -0.525 | 0.601 |  |
| BMMRS Forgiveness | -0.348 | -1.619 | 0.922 | 0.668 | -0.521 | 0.603 |  |
| BMMRS Negative Congregational Support | -0.054 | -1.847 | 1.605 | 0.844 | -0.064 | 0.949 |  |

Supplementary Table 6: This table reports model outputs for predicting morning closeness-to-God from all BMMRS factors. BMMRS = Brief Multidimensional Measure of Religion and Spirituality. Factor construction described in manuscript. * p<.05, ** p<.01, ***p<.001

**Supplementary Table 7: Mixed Effects Linear Regression Model Predicting Closeness-to-God from RPBS Factors**

|  | Estimate | 2.5_ci | 97.5_ci | SE | T-stat | P-val | Sig |
| --- | --- | --- | --- | --- | --- | --- | --- |
| (Intercept) | -6.284 | -9.472 | -3.452 | 1.486 | -4.229 | <.0001 | *** |
| Witchcraft | -0.282 | -0.963 | 0.465 | 0.347 | -0.815 | 0.417 |  |
| PSI | 0.302 | -0.662 | 1.272 | 0.477 | 0.634 | 0.527 |  |
| Superstition | 0.138 | -0.746 | 1.061 | 0.469 | 0.294 | 0.769 |  |
| Precognition | -0.891 | -1.815 | 0.06 | 0.479 | -1.859 | 0.065 | . |
| TRB | 1.191 | 0.718 | 1.654 | 0.225 | 5.289 | <.00010 | *** |
| Spiritualism | 1.673 | 0.732 | 2.573 | 0.485 | 3.453 | 0.001 | *** |

Supplementary Table 7: This table reports model outputs for predicting morning closeness-to-God from all Revised Paranormal Belief Scale (RPBS) factors. TRB = Traditional Religious Belief. * p<.05, ** p<.01, ***p<.001

**Supplementary Table 8. Mixed Effects Linear Regression Model Predicting Morning Closeness to God**

|  | Estimate | 2.5_ci | 97.5_ci | SE | T-stat | P-val | Sig |
| --- | --- | --- | --- | --- | --- | --- | --- |
| (Intercept) | -6.646 | -8.848 | -4.438 | 1.18 | -5.63 | <.0001 | *** |
| TRB | 0.123 | -0.326 | 0.609 | 0.224 | 0.55 | 0.583 |  |
| Spiritualism | 0.37 | -0.112 | 0.879 | 0.261 | 1.42 | 0.158 |  |
| BMMRS Positive Spirituality | 5.197 | 3.853 | 6.655 | 0.682 | 7.62 | <.0001 | *** |

Supplementary Table 8: Mixed-effects regression model predicting morning closeness-to-God from baseline measures selected from previous analysis. TRB = Traditional Religious Belief subscale of Revised Paranormal Belief Scale. BMMRS = Brief Multidimensional Measure of Religion and Spirituality. * p<.05, ** p<.01, ***p<.001

**Supplementary Table 9. Hyperparameter Combinations for ANN**

| **Model Hyperparameter** | **Values** |
| --- | --- |
| N (# perceptrons in node set) | 1, 2, 3, 4, 5, 6, 7, 8, 9, 10, 20 |
| Epochs | 250, 500, 750, 1000, 1250, 1500, 1750, 2000, 3000 |
| Optimizer | Root Mean Square Propagation (RMSProp) |
| Learning Rate | 0.001 |
| Activation Function | LeakyReLU |
| Scaling | Standard Scaling |

Supplementary Table 9. Hyperparameter combinations explored in sweep to determine best Temporal ANN.

**Supplementary Table 10. TSANN Hyperparameter Sweep for Closeness to God**

|  |  |  | **Temporal Neural Net** | | | | | | | |
| --- | --- | --- | --- | --- | --- | --- | --- | --- | --- | --- |
|  | **N** | **# of Reps** | **Closeness T-3 R2** | | **Closeness T-2 R2** | | **Closeness T-1 R2** | | **Closeness T R2** | |
| epochs |  |  | μ | std | μ | std | μ | std | μ | std |
| 250 | 3 | 30 | 0.129 | 0.061 | 0.183 | 0.059 | 0.237 | 0.1 | 0.216 | 0.078 |
| 500 | 3 | 30 | 0.347 | 0.088 | 0.516 | 0.068 | 0.636 | 0.061 | 0.618 | 0.056 |
| 750 | 3 | 30 | 0.505 | 0.06 | 0.624 | 0.055 | 0.69 | 0.064 | 0.687 | 0.056 |
| 1000 | 3 | 30 | 0.575 | 0.071 | 0.65 | 0.045 | 0.705 | 0.042 | 0.698 | 0.049 |
| 1250 | 3 | 30 | 0.638 | 0.054 | 0.674 | 0.041 | 0.718 | 0.05 | 0.734 | 0.041 |
| 1500 | 3 | 30 | 0.639 | 0.059 | 0.687 | 0.042 | 0.718 | 0.053 | 0.719 | 0.053 |
| 1750 | 3 | 30 | 0.646 | 0.06 | 0.658 | 0.051 | 0.687 | 0.056 | 0.699 | 0.049 |
| 2000 | 3 | 30 | 0.638 | 0.076 | 0.674 | 0.057 | 0.713 | 0.049 | 0.722 | 0.053 |
| 3000 | 3 | 30 | 0.64 | 0.047 | 0.649 | 0.044 | 0.668 | 0.052 | 0.692 | 0.047 |
| 250 | 6 | 30 | 0.214 | 0.057 | 0.475 | 0.048 | 0.604 | 0.051 | 0.578 | 0.052 |
| 500 | 6 | 30 | 0.421 | 0.067 | 0.614 | 0.038 | 0.684 | 0.04 | 0.687 | 0.043 |
| 750 | 6 | 30 | 0.568 | 0.049 | 0.675 | 0.047 | 0.73 | 0.04 | 0.728 | 0.043 |
| 1000 | 6 | 30 | 0.61 | 0.048 | 0.663 | 0.062 | 0.698 | 0.057 | 0.708 | 0.07 |
| 1250 | 6 | 30 | 0.621 | 0.057 | 0.674 | 0.055 | 0.705 | 0.052 | 0.721 | 0.048 |
| 1500 | 6 | 30 | 0.638 | 0.056 | 0.666 | 0.052 | 0.675 | 0.074 | 0.704 | 0.058 |
| 1750 | 6 | 30 | 0.66 | 0.057 | 0.686 | 0.049 | 0.691 | 0.048 | 0.702 | 0.048 |
| 2000 | 6 | 30 | 0.639 | 0.051 | 0.653 | 0.056 | 0.657 | 0.058 | 0.7 | 0.054 |
| 3000 | 6 | 30 | 0.614 | 0.081 | 0.628 | 0.065 | 0.597 | 0.086 | 0.659 | 0.075 |

Supplementary Table 10. Temporal Neural Net N = 3 hyperparameter variation results on Closeness-to-God.
